# Hyodeoxycholic acid attenuates atherosclerosis by antagonizing FXR and modulating the PD-1/mTORC1 signaling axis

**DOI:** 10.1016/j.redox.2026.104096

**Published:** 2026-02-21

**Authors:** Feng Yang, Wenqiong Huang, Zongzhen Meng, Meijun Liu, Aiping Lyu, Claudio Mauro, Kenneth C.P. Cheung

**Affiliations:** a Department of Cardiology, The First Affiliated Hospital, Zhejiang University School of Medicine, Hangzhou, Zhejiang Province, China; b Phenome Research Center, School of Chinese Medicine, Hong Kong Baptist University, Hong Kong, China; c Department of Inflammation and Ageing, College of Medicine and Health, University of Birmingham, Queen Elizabeth Hospital, Mindelsohn Way, B15 2WB, UK

**Keywords:** Atherosclerosis, Hyodeoxycholic acid, FXR, Regulatory T cells, Metabolism, Treg migration, Glycolysis, PD-1, mTORC1

## Abstract

Accumulating evidence suggested that bile acids play a significant role in modulating metabolic and inflammatory diseases. In this study, we investigated the roles of the farnesoid X receptor (FXR) and its endogenous antagonist hyodeoxycholic acid (HDCA) in the development of atherosclerosis (AS). We found that serum HDCA was significantly reduced in patients with AS, and systemic HDCA therapy attenuated plaque burden in vivo. Adoptive transfer of HDCA-treated Foxp3+ Tregs into ApoE-deficient recipients reduced lesion growth, whereas FXR-deficient Tregs failed to confer benefit. HDCA enhanced Treg migration and accumulation within plaques and reprogrammed Treg metabolism by antagonizing FXR and modulating PD-1/mTORC1 signaling. This shift relieved CPT1a-driven fatty acid oxidation bias, increased glycolysis and ATP production, and improved migratory capacity and effector function. We further identify ZNF671 as a transcriptional inhibitor of Treg migration that is mitigated by HDCA-dependent metabolic switching. Collectively, HDCA reduced FXR-mediated metabolic constraints while activating glycolytic and migratory programs in Tregs, thereby improving lipid handling and immune regulation within the plaque microenvironment. These findings position the HDCA–FXR–PD-1/mTORC1 axis as a novel immunometabolic target for AS.

## Highlights

• HDCA antagonizes FXR to reprogram Treg bioenergetics in atherosclerosis.
• HDCA shifts Tregs from FAO/OXPHOS to mTORC1-driven glycolysis.
• FXR antagonism lowers CPT1a, remodels mitochondria and lipid droplets.
• HDCA reduces PD-1/SHP-2 signaling, enhancing Treg migration into plaques.
• ZNF671 suppression and STAT5 activation further boost Treg function.

## Graphical abstract

**HDCA-driven FXR antagonism reprograms Treg metabolism and migration to inhibit atherosclerotic plaque growth.** The schematic summarizes how the bile acid hyodeoxycholic acid (HDCA) protects against atherosclerosis (AS) by acting directly on regulatory T cells (Tregs). Upper panel (in vivo overview, ApoE−/− AS blood vessel): HDCA promotes recruitment and infiltration of donor Foxp3+ Tregs into atherosclerotic plaques and reduces lipid deposition (fatty acids and cholesterol) within lesions. The protective effect depends on Treg-intrinsic FXR signaling being present and antagonized: HDCA-treated donor Tregs attenuate plaque development, whereas FXR-deficient Tregs do not confer benefit. HDCA also diminishes PD-1 signaling, which enhances Treg immune function and supports migration into the plaque microenvironment. Lower panel (inside Tregs, mechanistic view): HDCA antagonizes the Treg FXR receptor. This inhibition 1) downregulates the transcriptional repressor ZNF671, relieving its constraint on migratory gene programs; and 2) attenuates PD-1 signaling and its downstream suppression of mTORC1. The concerted blockade of these pathways initiates a metabolic switch: it suppresses FXR/CPT1a-driven fatty acid oxidation (FAO) and activates mTORC1, leading to enhanced glycolysis and ATP production. This metabolic reprogramming is further characterized by a global remodeling of the Treg metabolome, including altered amino acid flux and a shift toward unsaturated fatty acids. The resulting bioenergetic and biosynthetic surge fuels cytoskeletal reorganization, increases STAT5 phosphorylation, and enhances both the migratory capacity and suppressive functions of Tregs. Together, HDCA-driven FXR antagonism coordinates transcriptional, metabolic, and signaling changes that enhance Treg motility and function, leading to improved lipid handling and plaque stabilization.

**Figure:**
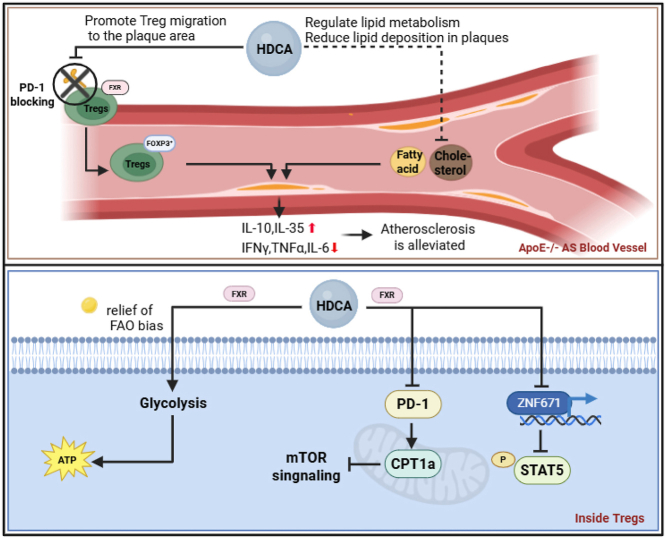

## 1 Introduction

Atherosclerosis (AS) remains a leading cause of cardiovascular morbidity and mortality globally, driven by complex interplay between lipid dysregulation and chronic vascular inflammation [1]. While immunometabolic crosstalk has been increasingly acknowledged as an important factor in AS pathogenesis, yet the molecular frameworks linking metabolic stress and dysfunctional immune responses are far from complete [2]. Among immune regulators, Foxp3^+^ regulatory T cells (Tregs) are indispensable for maintaining immune homeostasis, exerting potent immunosuppressive functions that prevent the development and progression of AS [3]. However, disease states are frequently marked by reduced Treg numbers and impaired suppressive capacity, attenuating their protective effects and allowing pathogenic immune responses to dominate within the arterial wall [4].

Recent advances have highlighted bile acids (BAs) as critical signaling molecules involved in systemic metabolism and immune cell function [5,6]. Beyond their classical roles in lipid absorption, BAs receptors such as farnesoid X receptor (FXR) and Takeda G protein-coupled receptor 5 (TGR5) orchestrate transcriptional programs crucial for cholesterol, triglyceride, and glucose homeostasis [7]. Disruption of these BA receptor signaling may exacerbate metabolic inflammation and the development of AS [8]. Notably, hyodeoxycholic acid (HDCA), a synthetic BA, has been identified as a potent FXR antagonist [9]. Our previous work has shown the therapeutic potential of HDCA on the regulation of glucose homeostasis and predicting type 2 diabetes risk [7,10]. In addition, HDCA enhances hepatic function by reducing lipid accumulation and lowering liver enzyme levels [11]. Clinical benefits have also been reported in primary biliary cholangitis and nonalcoholic steatohepatitis, where HDCA modulates BA metabolism and alleviates inflammation and fibrosis [12]. Moreover, emerging evidence implicates that BAs might play an important role in the onset and progression of AS [13,14].

Treg migratory is an active and energy-demanding process contingent upon dynamic metabolic reprogramming. In quiescent states, Tregs primarily utilize mitochondrial oxidative phosphorylation (OXPHOS) to meet their bioenergetic demands for homeostasis and suppressive function [15]. However, upon activation and recruitment, Tregs undergo a profound metabolic shift towards increased glycolysis, rapidly generate the substantial ATP and biosynthetic precursors required for cytoskeletal rearrangement, integrin activation, and sustained motility through challenging microenvironments [16]. This critical metabolic plasticity, governed by key sensors and pathways including HIF-1α and mTOR, ensures the cellular fuel and biosynthetic substrates necessary for effective migrating and subsequent local immunosuppression [17]. Accumulating evidence suggests that the migration of Tregs to atherosclerotic lesions can inhibit disease progression by regulating the local inflammatory and metabolic profile [18,19], Programmed death 1 (PD-1), an inhibitory receptor expressed on T cells, suppresses T cell activation and effector functions upon engagement with its ligands PD-L1 or PD-L2, primarily through recruitment of phosphatases such as SHP-2, which attenuate key downstream signaling pathways [20]. Among these, the mammalian target of rapamycin complex 1 (mTORC1) serves as a central regulator of T cell activation, metabolic reprogramming, proliferation, and differentiation [21]. Substantial evidence demonstrates that PD-1 signaling antagonizes mTORC1 activity, resulting in reduced glucose metabolism, inhibition of cell cycle progression, and promotion of T cell exhaustion [22]. In parallel, zinc finger (ZNF) proteins are emerging as pivotal modulators within the immune system, orchestrating the transcription of immune-related genes and thereby shaping the activation and functional properties of various immune cell subsets [23].

In this study, we delineate a previously unexplored immunometabolic circuit by which HDCA modulates Treg migration and function in AS. Through integrated clinical profiling, in vivo experimentation, adoptive transfer strategies, and comprehensive molecular analyses, we demonstrate that HDCA therapy mitigates atherosclerotic lesion burden, fosters Treg accumulation within inflamed vascular sites, and reprograms Treg metabolism by antagonizing FXR and orchestrating the PD-1/mTORC1 signaling axis. These findings establish the HDCA–FXR–PD-1/mTORC1 axis as a vital pathway for Treg migration, uncovering novel therapeutic targets for AS treatment.

## 2 Results

### 2.1 HDCA improves the pathological features of AS and enhances the infiltration of Tregs into plaques

**Fig. 1:**
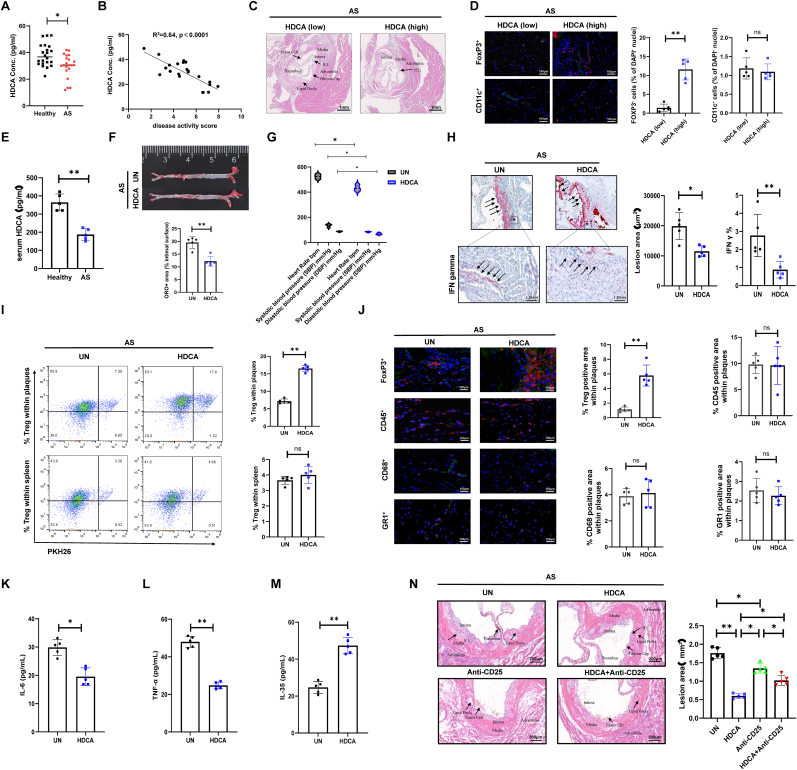
**AS was associated with decreased serum levels of HDCA and impaired Treg accumulation within vascular plaques. (A)** Serum HDCA levels were reduced in AS patients relative to healthy individuals. The observed overlap reflects expected biological variability in human populations. **(B)** A negative correlation was observed between serum HDCA concentration and disease activity scores in AS patients. **(C)** Histopathological analysis of arterial samples revealed that higher HDCA levels correlated with reduced arterial intima thickness and plaque formation (magnification, 2 × ; scale bar, 1 mm). **(D)** Confocal microscopy showed increased infiltration of Foxp3^+^ Tregs (red) in AS patients with higher HDCA levels, whereas the percentages of CD11c^+^ cells (red) did not significantly differ between groups. CD25^+^ cells (green, in panel A) and MHC-II^+^ cells (green, in panel B) are presented for additional immunofluorescent phenotyping. Cell nuclei were stained with DAPI (blue). Representative images are shown (magnification, 10 × ; scale bar, 100 μm). **(E)** Serum HDCA concentrations are significantly decreased in AS mouse models. **(F)** Oil Red O staining of arterial sections showing reduced lipid deposition following HDCA treatment. **(G)** Assessment of hemodynamic parameters, including systolic and diastolic blood pressure (SBP and DBP) and heart rate. **(H)** Oil Red O and immunohistochemical analysis of lesions area and IFN-γ expression in arterial section (magnification, 2 × ; scale bar, 1.25 mm). **(I)** Flow cytometry of PKH26-labeled donor Tregs revealed that HDCA treatment selectively increased Treg accumulation in atherosclerotic plaques, with no effect in spleen tissue. **(J)** Quantification of Foxp3^+^ Tregs (red), CD45^+^ leukocytes (red), CD68^+^ macrophages (red), and GR1^+^ monocytes/granulocytes (red) in atherosclerotic plaques following HDCA treatment; approximately 500 cells were counted per group. CD25^+^ cells (green), CD3^+^ T cells (green), F4/80^+^ macrophages (green), and Ly6G^+^ neutrophils (green) were visualized by immunofluorescence co-staining. Cell nuclei were labeled with DAPI (blue). **(K**–**L)** ELISA quantification of serum IL-6 (K) and TNF-α (L) levels in AS mice with or without HDCA treatment. **(M)** IL-35 levels in the supernatant of Treg cells were measured by ELISA after 48 h of culture with or without HDCA treatment. **(N)** Representative H&E staining of arterial sections from AS mice to evaluate atherosclerotic lesion area following HDCA or anti-CD25 treatment. Data are presented as mean ± SD (n = 5 biological replicates). Statistical significance was determined using two-tailed Student's *t*-test or one-way ANOVA. ns: not significant; ∗*P* < 0.05, ∗∗*P* < 0.01, ∗∗∗*P* < 0.001.

We conducted an analysis of blood samples from 43 participants, including 21 AS patients and 22 healthy donors (HD), to investigate the association between serum HDCA levels and AS. Serum HDCA concentrations were quantified by ultra-performance liquid chromatography–tandem mass spectrometry (UPLC–MS/MS) using an isotopically labeled internal standard. Our findings indicate a trend toward lower serum HDCA concentrations in AS patients compared to HD and were negatively correlated with disease activity scores in the AS cohort (Fig. 1A and B). Despite the inter-patient variability within cohorts, a common feature of human metabolic profiles [24], this trend aligns with the functional impairments observed in our experimental models and suggests a potential link that warrants further investigation. H&E staining showed that AS patients with higher serum HDCA had reduced arterial intima thickness and plaque burden (Fig. 1C). Furthermore, immunostaining revealed high HDCA levels were associated with an increased number of Foxp3+ Tregs infiltration within the plaques, whereas the infiltration of CD11c + macrophage/dendritic cells remained unchanged, indicating that elevated HDCA specifically promotes Treg accumulation without affecting CD11c + cell recruitment (Fig. 1D).

We next evaluated the effects of HDCA in APOE−/− mice with AS induced by a high-fat diet (HFD). Consistent with our clinical results, serum HDCA levels were reduced in AS mice compared to healthy controls (Fig. 1E). Treatment with HDCA (10 mg/kg/day, for 6 weeks) significantly reduced lipid deposition within arterial plaques (Fig. 1F) and improved hemodynamic parameters, including heart rate, systolic and diastolic blood pressure (Fig. 1G). In addition, HDCA treatment was associated with reduced plaque area and lower levels of local inflammatory markers, such as IFN-gamma (IFN-γ) (Fig. 1H). Notably, HDCA increased the recruitment of Tregs to the plaques without affecting their abundance in the spleen, indicating that HDCA specifically promotes Treg migration to the lesion site (Fig. 1I). To further exclude potential off-target effects on other immune cell populations in vivo, we evaluated the infiltration of leukocytes (CD45^+^), macrophages (CD68^+^), and monocytes/granulocytes (GR1+) using immunostaining, and observed no significant changes in these populations (Fig. 1J).

Furthermore, HDCA administration markedly suppressed systemic inflammation, as evidenced by decreased serum concentrations of pro-inflammatory cytokines IL-6 and TNF-α (Fig. 1K and L). To further evaluate the functional consequences of the phenotypic changes induced by HDCA, we assessed the suppressive capacity of Tregs. HDCA-treated Tregs exhibited significantly increased production of the immunosuppressive cytokine IL-35, along with upregulated expression of the ATP-degrading ectoenzymes CD73 and CD39, confirming an enhanced regulatory phenotype (Fig. 1M, Fig. S1A–B). To verify the stability of the Treg lineage, we next examined the co-expression of FoxP3 with the inflammatory cytokines IL-17 and IFN-γ within the Treg compartment. HDCA treatment significantly reduced the proportions of FoxP3+IL-17+ and FoxP3+IFN-γ+ cells (Fig. S1C–D), indicating a robust maintenance of Treg stability and a decreased propensity for conversion towards pro-inflammatory phenotypes.

To definitively establish whether the protective effects of HDCA are mediated by Treg cells, APOE−/− mice were treated with an anti-CD25 antibody to selectively deplete Tregs in vivo. Histological assessment of arterial sections revealed that HDCA treatment markedly reduced atherosclerotic lesion area compared to untreated controls, whereas Treg depletion by anti-CD25 abrogated this effect (Fig. 1N). Flow cytometric analysis demonstrated that anti-CD25 administration efficiently depleted CD4^+^Foxp3^+^ Tregs within the aortic plaque, resulting in significantly lower Treg frequencies in the lesion site (Fig. S1E). In parallel, measurement of serum total cholesterol showed that the reduction induced by HDCA was reversed upon Treg depletion, indicating that Treg cells as a key determinant of HDCA-induced cholesterol reduction (Fig. S1F). These findings highlight HDCA's protective effects on cardiovascular function and its capacity to mitigate arterial inflammation and lesion formation, potentially via regulating Treg infiltration within the plaque area.

## 3 FXR-dependent Treg modulation underlies HDCA-mediated atheroprotection

**Fig. 2:**
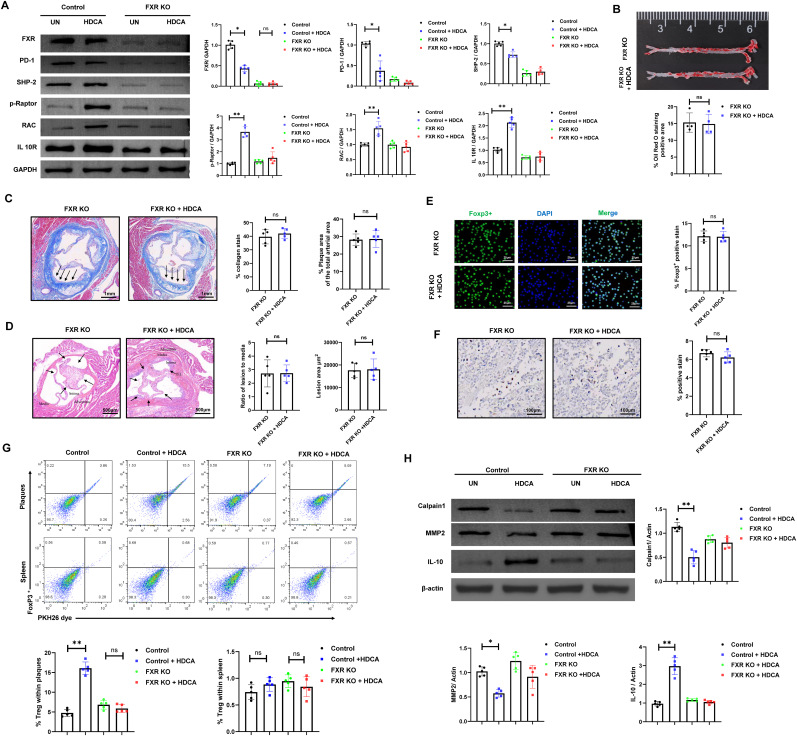
**HDCA modulates Treg migration and atherosclerotic plaque composition via FXR signaling.** ApoE−/− mice (C57BL/6J background, male, 8 weeks old) were fed a high-fat diet for 28 days to induce AS and subsequently received adoptive transfer of control or FXR-knockout (FXR KO) Treg cells generated by CRISPR/Cas9-mediated lentiviral transduction, with HDCA (30 μM) or vehicle treatment as indicated. **(A)** Representative Western blot analysis of FXR, PD-1, SHP-2, p-Raptor, RAC and IL-10R expression in isolated Treg cells from each group. **(B)** Oil Red O staining was performed to assess lipid accumulation in the aorta. **(C)** Masson's trichrome staining of aortic sections from FXR KO mice reveals comparable plaque area and collagen deposition in both HDCA-treated and untreated groups (magnification, 5 × ; scale bar, 1 mm). **(D)** Representative H&E images of aortic sections show the difference between untreated and HDCA-treated mice in the FXR KO groups (magnification, 5 × ; scale bar, 500 μm). Relative bar graphs show quantification of lesion area and lesion/media area ratio. **(E)** Confocal immunofluorescence was used to evaluate Foxp3+ Treg infiltration within atherosclerotic plaques (scale bar, 25 μm). **(F)** Immunohistochemistry images show Treg accumulation in the plaque area across all groups (magnification, 40 × ; scale bar, 100 μm). **(G)** Flow cytometry analysis of Treg proportions in aortic plaques and spleen from control and FXR KO mice, with or without HDCA treatment. **(H)** Western blot analysis of matrix remodeling-related proteins, including calpain 1 and matrix metalloproteinase 2, and the anti-inflammatory factor IL-10. Data are presented as mean ± SD (n = 5 biological replicates). Data with four groups were analyzed by one-way ANOVA with Tukey's post hoc test. Comparisons between two groups were performed using the non-parametric Mann-Whitney *U* test. ns, not significant; ∗*P* < 0.05, ∗∗*P* < 0.01, ∗∗∗*P* < 0.001.

Previous studies have identified HDCA targets FXR receptor, capable of modulating metabolic diseases through FXR-dependent pathways [5,7]. To further investigate whether the immunomodulatory effects of HDCA depend on the presence of FXR, donor Tregs were subjected to FXR gene knockout using CRISPR/Cas9-mediated lentiviral transduction, then treated with HDCA prior to adoptive transfer into HFD-induced atherosclerotic recipient mice for functional assessment. Western blot analysis demonstrated that HDCA treatment in control Tregs not only downregulated FXR expression, but also decreased the expression of PD-1 and its downstream effector SHP-2, while upregulating phosphorylated Raptor (p-Raptor), RAC, and IL-10R. This pattern suggests that HDCA promotes Treg activation and a shift toward a phenotype with enhanced migratory capacity and immunoregulatory function. Mechanistically, suppression of PD-1/SHP-2 signaling is known to relieve constraints on TCR-induced mTOR activation and glycolytic metabolism in T cells, thereby supporting cells migration, and functional activity [25]. In contrast, in FXR KO Treg, the basal expression of these proteins did not change significantly following HDCA treatment, indicating that the HDCA-induced phenotypic reprogramming of Treg requires the presence of FXR (Fig. 2A).

Evaluation of AS outcomes demonstrated that HDCA-treated FXR KO Tregs did not reduce aortic lipid plaque burden relative to FXR KO controls (Fig. 2B). Quantification of aortic sections stained with Masson's trichrome and hematoxylin & eosin revealed no significant differences in overall lesion area and collagen deposition between HDCA-treated and untreated mice (Fig. 2C and D). Immunofluorescence and immunohistochemistry analyses of CD4^+^Foxp3^+^ Tregs revealed that HDCA treatment did not alter Treg infiltration in FXR KO mice, a result further supported by flow cytometric assessment of Treg migration within atherosclerotic plaques and spleen (Fig. 2E–G). By contrast, HDCA administration markedly increased Treg migration into plaques in the presence of FXR (Fig. 2G). In parallel experiments, Western blot analysis of arterial tissue confirmed that treated with HDCA significantly downregulated matrix remodeling-related proteins, such as Calpain1 and MMP2, while upregulating the anti-inflammatory factor IL-10; an effect that was abolished in FXR-deficient mice (Fig. 2H). These results further suggest that HDCA promotes plaque stabilization and anti-inflammatory responses in an FXR-dependent manner.

To further substantiate the requirement for FXR, we assessed the impact of HDCA in FXR-overexpressing (FXR OE) mice. HDCA treatment in FXR OE mice exhibited significantly reduced aortic lipid deposition and lesion area (Fig. S2A–B). Moreover, Foxp3+ immunofluorescence and flow cytometric quantification revealed a pronounced increase in Treg infiltration within the plaques of HDCA-treated FXR OE mice, whereas splenic Treg frequencies remained unchanged (Fig. S2C–D). These data collectively indicate that HDCA confers atheroprotection require FXR, and that its immunomodulatory effects in promoting plaque-resident Treg migration and mitigating lesion development are mediated through inhibition of FXR.

In addition, to gain further insight into the cellular and molecular mechanisms underlying the effects of HDCA, comprehensive in vitro studies were performed. FXR reporter assays demonstrated that HDCA markedly inhibited GW4064-induced FXR transcriptional activity in a dose-dependent manner (Fig. S2E–G), and ChIP-qPCR analysis confirmed that GW4064 treatment robustly increased FXR binding to Nr0b2 and CPT1a promoter regions, while this enrichment is significantly reduced by HDCA co-treatment, indicating inhibition of FXR recruitment to target genes (Fig. S2H). Functional assays demonstrated that HDCA enhanced cytoskeletal remodeling in Tregs, indicated by increased F-actin polymerization and elevated RAC1-GTP levels—an upstream regulator of cell motility—effects that were reversed by FXR agonism (Fig. S2I–J). Consistent with these findings, GW4064 effectively abrogated the HDCA-induced enhancement of Treg chemotactic migration in vitro (Fig. S2K), further demonstrating that the promigratory effect of HDCA is dependent on FXR inhibition and establishing FXR as a key negative regulator of Treg migratory capacity.

## 4 In vitro HDCA treatment shifts the metabolic profile of Tregs

The accumulation and enhanced migratory capacity of Tregs are critical determinants in the modulation of immune dynamics within atherosclerotic lesions [26]. Although the immunosuppressive roles of Tregs have been extensively characterized, the metabolic mechanisms underpinning their increased mobility remain largely elusive. To explore the link between metabolic reprogramming and Treg migration, we performed a series of metabolic flux analyses, focusing on the regulatory role of the HDCA: FXR axis in modulating Treg metabolism and mitochondrial remodeling.

**Fig. 3:**
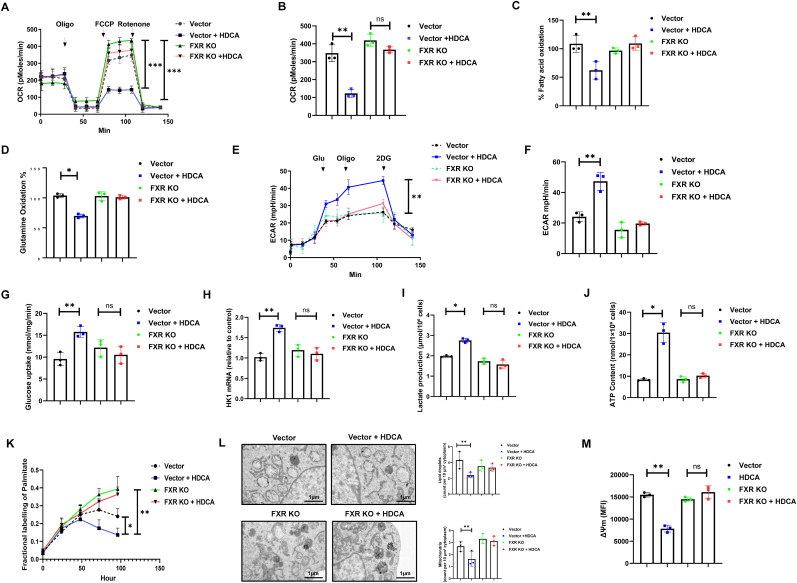
**HDCA–FXR axis orchestrates mitochondrial remodeling and metabolic flexibility in Tregs. (A**–**B)** The OCR measurements indicated that HDCA treatment decreased OCR in vector control Tregs, while FXR KO Tregs showed no significant change. **(C**–**D)** Assessment of FAO and glutathione oxidation in the vector control group compared to FXR KO Tregs ± HDCA. (**E-F)** ECAR analysis revealed that HDCA treatment significantly enhanced glycolytic activity in vector control Tregs. **(G**–**I)** Glucose uptake (G), HK1 mRNA expression (H), and lactate production (I) in vector and FXR KO Tregs with or without HDCA treatment. **(J)** Cellular ATP content in vector and FXR KO Tregs ± HDCA. **(K)** Fractional labeling of palmitate, assessed by ^13^C-glucose incorporation and mass spectrometry. **(L)** Transmission electron microscopy (TEM) images reveal decreased accumulation of lipid droplets and mitochondria after HDCA treatment in vector controls (scale bar, 1 μm). **(M)** Measurement of mitochondrial membrane potential in Tregs under indicated conditions. Data are presented as mean ± SD (n = 3 biological replicates). Statistical significance was determined using one-way ANOVA with Tukey's post hoc test. ns, not significant; ∗*P* < 0.05, ∗∗*P* < 0.01, ∗∗∗*P* < 0.001.

Our findings demonstrate that HDCA treatment significantly decreased the oxygen consumption rate (OCR) in control Tregs, indicative of inhibited mitochondrial respiration. Notely, HDCA exposure did not further reduce OCR in FXR-KO Tregs, confirming the essential role of FXR in regulating Treg metabolism in response to HDCA (Fig. 3A and B). Assessment of substrate oxidation demonstrated a marked decrease in the percentage of fatty acid oxidation (Fig. 3C) and glutamine oxidation rates (Fig. 3D), indicating that a metabolic impairment in mitochondrial fuel utilization upon FXR deficiency. In response, Tregs exhibited enhanced glycolytic activity, as evidenced by increased extracellular acidification rates (ECAR), elevated glucose uptake, and upregulation of the glycolytic enzyme HK1 (Fig. 3E–H). Notably, this glycolytic reprogramming was further associated with increased lactate secretion, creating a more acidic intracellular environment that may modulate cellular function and enhance their migratory capacity [27] (Fig. 3I). Furthermore, HDCA treatment led to a transient elevation of intracellular ATP levels in the presence of FXR, thereby supplying the necessary energy to support immune cell migration [28] (Fig. 3J).

To investigate fatty acid utilization by Tregs, we performed a palmitate-labeling experiment, which revealed decreased fractional labeling of palmitate following HDCA treatment, indicative of impaired uptake and/or oxidation of exogenous fatty acids (Fig. 3K). In atherosclerotic lesions, lipid droplets (LDs) accumulate in response to elevated local fatty acid concentrations, and serve as essential energy reservoirs for infiltrating immune cells [29]. Tregs specifically utilize LDs by mobilizing stored fatty acids for ATP production through fatty acid oxidation (FAO) [30]. Intriguingly, we observed a pronounced decreases in both LD and mitochondrial content, together with a significant loss of mitochondrial membrane potential in Tregs exposed to HDCA (Fig. 3L and M), consistent with our previous observation that HDCA inhibits FAO. Taken together, these findings highlight the crucial role of FXR signaling in maintaining mitochondrial integrity and metabolic homeostasis, and further demonstrate that inhibition of FXR disrupts cellular energy metabolism primarily through suppression of mitochondrial fatty acid oxidation.

## 5 HDCA–FXR axis regulates lipid homeostasis and inflammation in AS

**Fig. 4:**
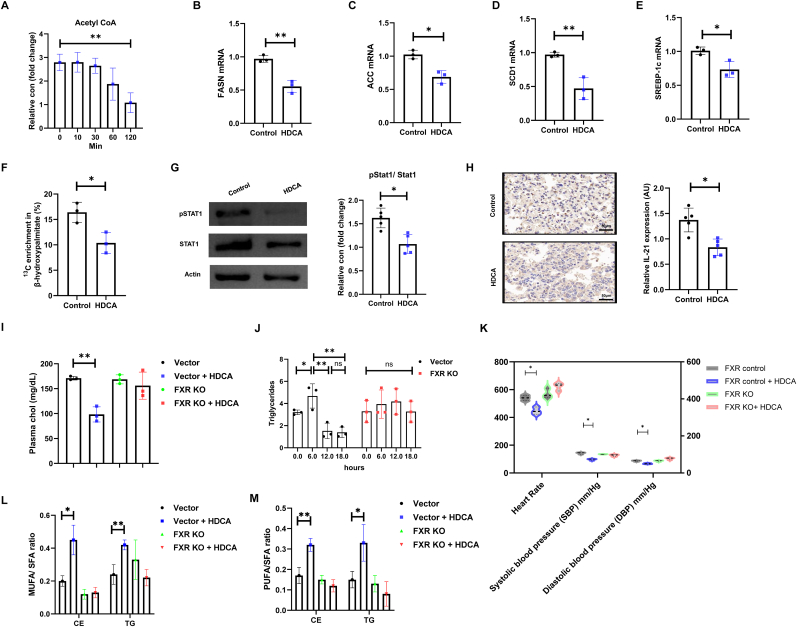
**HDCA-treated ameliorate inflammation and modulate lipid metabolism in vivo. (A)** Acetyl-CoA levels decreased over time in HDCA-treated Tregs. **(B**–**E)** Quantitative real-time PCR analysis of the mRNA expression of key lipogenic genes, including (B) fatty acid synthase (FASN), (C) acetyl-CoA carboxylase (ACC), (D) SCD1, and (E) SREBP-1c, in Tregs with or without HDCA treatment. **(F)** Functional metabolic flux assay using ^13^C-glucose tracing shows decreased enrichment of labeled β-hydroxypalmitate in the HDCA-treated group. **(G)** Immunoblot analysis of phosphorylated STAT1 in Tregs following HDCA exposure, and quantification statistical analysis results are presented in the bar graphs. **(H)** Representative IHC images show reduced IL-21 expression in atherosclerotic lesions of mice following HDCA treatment (magnification, 5 × ; scale bar, 50 μm). **(I)** Plasma cholesterol levels were measured by enzymatic colorimetry assay in FXR-sufficient controls and FXR KO mice ± HDCA. **(J)** Plasma triglyceride levels in the Vector and FXR KO groups were quantified at 0, 6, 12, and 18 h by a colorimetric assay. **(K)** Hemodynamic profiling assessed heart rate, systolic blood pressure (SBP), and diastolic blood pressure (DBP) for each group. **(L**–**M)** Ratios of monounsaturated to saturated fatty acids (MUFA/SFA) and polyunsaturated to saturated fatty acids (PUFA/SFA) in serum cholesterol esters (CE) and triglycerides (TG) were quantified by lipidomics in FXR-sufficient control and FXR KO mice ± HDCA. Data are presented as the mean ± SD (n = 3-5 biological replicates). ∗*P* < 0.05, ∗∗*P* < 0.01.

To further assess the effect of HDCA on Treg lipid metabolism, we first quantified acetyl-CoA levels following HDCA treatment over a time course. Our results demonstrate that HDCA administration significantly reduced intracellular acetyl-CoA levels, with the most notable decrease observed at 120 min, indicating a rapid suppression of the key substrate for fatty acid synthesis (Fig. 4A). Concomitantly, HDCA treatment significantly downregulated the mRNA expression of key lipogenic enzymes such as fatty acid synthase (FASN), acetyl-CoA carboxylase (ACC) and SCD1, as well as the central transcriptional regulator SREBP-1c, demonstrating that HDCA inhibits both the enzymatic and transcriptional machinery governing lipid biosynthesis (Fig. 4B–E). Consistent with these findings, functional metabolic flux assays using ^13^C-glucose incorporation demonstrated a reduced proportion of labeled β-hydroxy-palmitate, further substantiating the inhibitory effect of HDCA on Treg de novo lipogenesis (Fig. 4F).

In addition, to better understand immunomodulatory activity of HDCA, we analyzed inflammatory markers in Tregs. Western blot analyses revealed that HDCA lowers phosphorylation of STAT1 without affecting total STAT1 protein levels, suggesting targeted interference with STAT1 activation (Fig. 4G). Consistently, immunohistochemical staining illustrated that HDCA robustly suppresses the expression of IL-21, a pro-inflammatory cytokine regulated by STAT1 signaling, reflecting HDCA's capacity to attenuate aortic inflammation through regulation of STAT1-mediated pathways (Fig. 4H).

We next assessed FXR dependence of these effects to elucidate their impact on metabolic and cardiovascular phenotypes. Systemically, HDCA administration led to a substantial reduction in plasma cholesterol and triglyceride levels in the control group, while these beneficial effects were markedly attenuated in FXR-deficient mice, underscoring the critical role of FXR in mediating HDCA-induced metabolic regulation (Fig. 4I and J). Notably, HDCA exhibited a time-dependent effect on triglyceride metabolism, showing a transient increase at 6 h, followed by a sustained reduction at 12 and 18 h. This biphasic pattern suggests an initial phase of lipid mobilization or metabolic adaptation preceding the longer-term lipid-lowering effect [31]. Hemodynamic profiling showed that HDCA treatment significantly decreased heart rate and blood pressure in control but not FXR-deficient mice, suggesting FXR-mediated systemic or vascular effects that may secondarily influence lipid metabolism (Fig. 4K). Lipidomic analysis revealed that HDCA treatment led to increased unsaturation, as evidenced by higher ratios of polyunsaturated and monounsaturated fatty acids to saturated fatty acids (PUFA/SFA and MUFA/SFA) in both cholesteryl ester and triglyceride fractions (Fig. 4L and M). While elevated PUFA/SFA ratios are generally associated with anti-inflammatory and atheroprotective lipid profiles [32], such favorable changes were not observed in FXR KO mice, as HDCA failed to elevate either MUFA/SFA or PUFA/SFA ratios in the absence of FXR.

Furthermore, HDCA-induced metabolic improvements were accompanied by structural and immunological changes within atherosclerotic plaques. Specifically, HDCA treatment increased COL1A1 deposition, indicative of enhanced fibrous cap stability, and reduced MMP2 expression, a key mediator of extracellular matrix degradation and plaque instability. These shifts suggest that HDCA promotes plaque stabilization and reduces rupture risk through FXR-dependent remodeling of the vascular matrix (Fig. S3A). Additionally, HDCA augmented lysosome abundance, reflecting heightened autophagic and degradative capacity that facilitates the clearance of lipids and inflammatory debris, thereby contributing to overall vascular homeostasis and a reduction in atherosclerotic burden [33] (Fig. S3B). Collectively, these results delineate a comprehensive role for HDCA in modulating lipid profiles and tempering inflammation in AS, with the HDCA–FXR axis serving as a central regulator during AS progression.

## 6 HDCA synergizes with PD-1 blockade to optimize Treg metabolic fitness and protective capacity via mTORC1

**Fig. 5:**
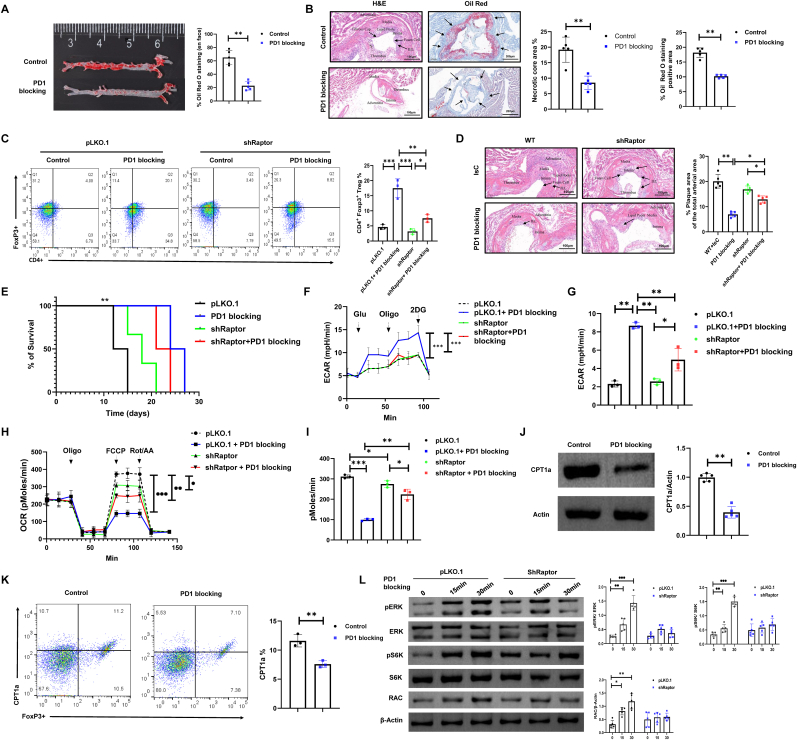
**PD-1 blockade promotes Treg migration via mTORC1-driven glycolytic pathways**. **(A)** Representative en face images of Oil Red O-stained aortas from control and PD-1 blockade groups. **(B)** Representative images of arterial lesions stained with H&E (scale bar, 100 μm) and Oil Red O (scale bar, 200 μm), with quantification of the necrotic core area and Oil Red O-positive areas. **(C)** Flow cytometry detection of the enrichment of Foxp3+ Tregs in plaque areas following adoptive transfer of Tregs transduced with shRaptor or control vector, with or without PD-1 blockade. **(D)** Representative H&E staining of aortic sections showing the percentage of atherosclerotic plaque area relative to the total arterial area in each group (scale bar, 100 μm; magnification, 10 × ). **(E)** Kaplan-Meier survival analysis of skin grafts in recipients treated with control Tregs (pLKO.1), shRaptor Tregs, PD-1 blockade, or shRaptor plus PD-1 blockade. **(F–I)** Metabolic analysis of donor Tregs after shRaptor or PD-1 blockade, showing effects on glycolysis (ECAR) and mitochondrial respiration (OCR). **(J**–**K)** Western blot and flow cytometry analysis of CPT1a expression levels in Treg isolated from plaques to evaluate alterations in fatty acid oxidation pathways following PD-1 blockade. **(L)** Western blot analysis was used to assess pERK, ERK, pS6K, S6K, and RAC levels in Treg cells transduced with control shRNA (pLKO.1) or Raptor shRNA (shRaptor) following PD-1 blockade at 0, 15, and 30 min. Statistical analyses were performed using one-way ANOVA followed by Tukey's post-hoc test for multiple comparisons, and the log-rank test for survival analysis. Data are presented as mean ± SD (n = 3-5 biological replicates). Statistical significance is indicated as follows: ∗*P* < 0.05, ∗∗*P* < 0.01, ∗∗∗*P* < 0.001.

Our previous data have suggested that HDCA-induced decrease in PD-1 and FXR protein in plaque-detached Treg was significantly correlated with HDCA-induced reduction in Sirius red staining area and the increases in cardiac fractional shortening (Fig. S4A–D), suggesting that HDCA exerts a beneficial effect on vascular remodeling and cardiac function, potentially through suppression of PD-1 and FXR. In addition, immunofluorescence analysis further confirmed that HDCA treatment significantly downregulated PD-1 and FXR in lesional Tregs (Fig. S4E). While the metabolic regulation of Tregs by HDCA has been established to depend on FXR, the contribution of PD-1 to this process remains largely undefined. To address whether PD-1 inhibition is involved on the therapeutic effects of HDCA. We conducted experiments using ex vivo expanded donor Treg that subjected to PD-1 blockade or received isotype-matched controls prior to intravenous injection into syngeneic mice with AS. The results indicated that PD-1 blockade significantly reduced Oil Red O staining and necrotic core areas in recipient AS mice compared to those that received control Tregs (Fig. 5A and B).

Given mTORC1's pivotal function as a metabolic orchestrator in Treg cells and negative regulation by PD-1 signaling [34], we next investigate the involvement of mTORC1 on PD-1 signaling. Using shRNA-mediated Raptor knockdown, we genetically disrupted mTORC1 signaling in Tregs and assessed their response to PD-1 blockade. Flow cytometry analysis revealed PD-1 blockade increased Treg accumulation within plaques, while an effect that was significantly diminished upon Raptor knockdown, implicating mTORC1 signaling is essential for the accumulation of Tregs within atherosclerotic plaques in response to PD-1 blockade (Fig. 5C). Consistently, histological assessment of plaque burden further demonstrated that PD-1 blockade induced atheroprotection is abrogated upon Raptor knockdown (Fig. 5D). To extend these observations to alloimmunity, we used a fully MHC-mismatched skin transplant rejection model (BALB/c skin grafted onto C57BL/6 recipients), which serves as a rigorous and clinically relevant system for assessing Treg migration and functional capacity in vivo [35]. Our findings confirmed that adoptively transferred shRaptor Tregs exhibited diminished the prolongation of graft survival conferred by PD-1 blockade (Fig. 5E).

Extracellular flux analysis revealed that PD-1 blockade substantially enhanced glycolytic activity in Tregs, as evidenced by increased ECAR. However, mTORC1-deficient Tregs (shRaptor) exhibited a profound glycolytic defect, and the stimulatory effect of PD-1 blockade was largely absent in this group (Fig. 5F and G). Measurements of OCR demonstrated that basal and maximal mitochondrial respiration was significantly downregulated following PD-1 blockade in pLKO.1 Tregs, but this was markedly increased in shRaptor Tregs (Fig. 5H and I), supporting an essential role for mTORC1 in metabolic reprogramming induced by PD-1 inhibition. Moreover, we found that PD-1 blockade suppresses CPT1a, a rate-limiting enzyme in fatty acid metabolism (Fig. 5J and K), indicative of a shift that may facilitate the metabolic adaptation necessary for optimal Treg function [36]. At the signaling level, PD-1 blockade significantly enhances phosphorylation of ERK and S6K, canonical readouts of MAPK and mTORC1 activity, respectively [37,38]. Moreover, PD-1 inhibition upregulates RAC expression, a small GTPase critically involved in cytoskeletal reorganization and Treg migration [35]. Notably, these signaling responses were entirely lost in Raptor-deficient Tregs, further emphasizing the essential requirement for mTORC1 in mediating the metabolic and functional consequences of PD-1 blockade (Fig. 5L).

To further investigate whether PD-1 inhibition is involved on the therapeutic effects of HDCA, we first assessed the expression of PD-1 and Raptor in Treg cells. Flow cytometric analysis revealed that HDCA treatment robustly decreased PD-1 surface expression and concomitantly increased Raptor levels compared to vector controls, indicative of enhanced mTORC1 activity (Fig. S4F–G). In parallel, a marked increase in the proportion of CD4^+^Foxp3^+^ Tregs was observed, reflecting enhanced Treg infiltration and migratory capacity (Fig. S4H). Metabolic profiling demonstrated that both HDCA and PD-1 blockade significantly elevated ECAR while concomitantly reducing OCR, consistent with a metabolic shift toward aerobic glycolysis and away from mitochondrial respiration in Treg cells; with the combined intervention amplifying these effects (Fig. S4I–J). In line with these metabolic changes, pERK and pS6K were upregulated by either HDCA or PD-1 blockade (Fig. S4K–L). Furthermore, enhanced production of anti-inflammatory cytokines IL-10 and IL-35 was also observed in these groups (Fig. S4M–N). Collectively, these data establish that HDCA exerts its immunometabolic benefits through suppression of PD-1 and activation of mTORC1, reprogramming Treg metabolism and enhancing overall immune response.

## 7 Regulatory role of ZNF671 in HDCA/FXR-mediated Treg cell metabolic and migratory adaptation in AS

Zinc (Zn) is an essential molecule for the function of many intracellular proteins. It is well-accepted that Zn homeostasis plays a pivotal role on immune cell metabolism and differentiation [39]. Notably, the activation of FXR/SHP is responsible for the reduction of lipid content on yellow catfish hepatocytes mediated by Zn [40]. Accumulating evidence in cancer cells suggest zinc finger protein 671 (ZNF671) play an important role in cell differentiation, proliferation, apoptosis and tumor suppression [41], while its role in Tregs remain elusive.

**Fig. 6:**
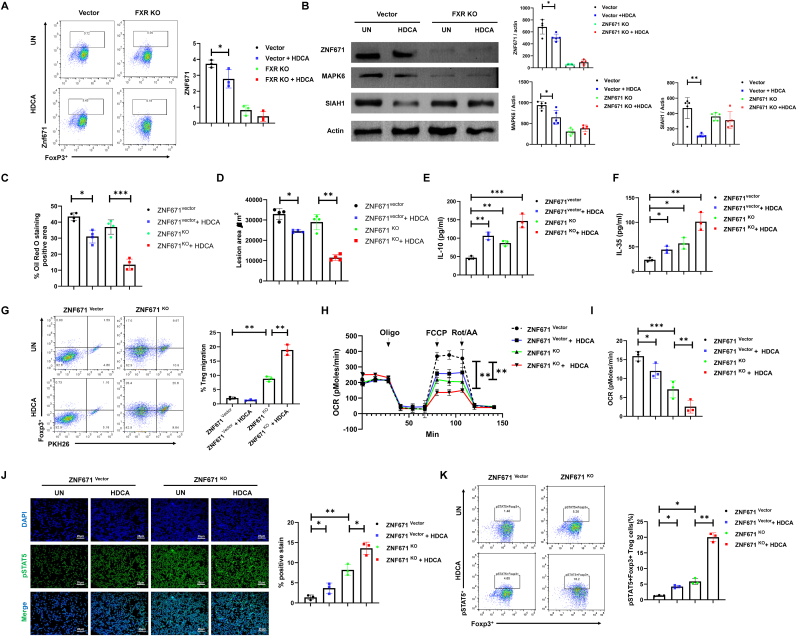
**ZNF671 governs HDCA-driven STAT5 activation and metabolic adaptation in Treg cells.** Ex vivo Tregs were transduced with shZNF671 or vector treated w/wo HDCA prior to iv-injection in recipient AS recipients. Tregs isolated from atherosclerotic plaque based on Foxp3 expression. **(A)** Flow cytometric analysis and quantification ZNF671 expression in Foxp3+ Tregs isolated from plaques in Vector and FXR KO groups ± HDCA. **(B)** Western blot analysis of protein expression levels of ZNF671, MAPK6, and SIAH1 in each group. **(C)** Quantification of atherosclerotic plaque area by percentage of Oil Red O staining-positive area in aorta from different groups. **(D)** Quantification of atherosclerotic lesion area in indicated groups. **(E**–**F)** ELISA was used to quantify the levels of anti-inflammatory cytokines IL-10 and IL-35 in culture supernatants of Treg cells from the indicated groups. **(G)** Migration of PKH26-labeled Treg cells was quantified by flow cytometry 24 h post-transfer. **(H–I)** Seahorse assay showed that OCR was significantly reduced in the ZNF671 KO group and this reduction was further exacerbated by HDCA treatment. **(J)** Immunofluorescence staining showed that ZNF671 KO enhanced pSTAT5 signaling, and this effect was significantly amplified by HDCA treatment. Quantification of pSTAT5 levels is shown in the bar graphs as mean ± SD from 500 cells per coverslip. scale bar, 25 μm. **(K)** Flow cytometry plots and quantification the percentage of pSTAT5+Foxp3+ Treg cells across all groups. Data are presented as the mean ± SD (n = 3-5 biological replicates). Statistical analysis was performed using one-way ANOVA followed by post-hoc tests. ∗*P* < 0.05, ∗∗*P* < 0.01, ∗∗∗*P* < 0.001.

We first assessed the impact of HDCA and FXR signaling on ZNF671 expression, our results showed that HDCA significantly decreased ZNF671 expression in control Tregs, whereas FXR-deficient Tregs exhibited substantially lower baseline ZNF671 levels that was unresponsive to HDCA (Fig. 6A). In parallel, HDCA downregulated MAPK6 and SIAH1 in control Tregs, while their expression remained low or unaltered in FXR KO Tregs following HDCA exposure (Fig. 6B), indicating that intact FXR signaling is required for HDCA-mediated modulation of these targets in Tregs. These findings align with previous reports that MAPK pathway inhibition can promote Treg differentiation and tissue protection, and that perturbations in SIAH family E3 ligases influence Treg cell cycle and tissue infiltration [42,43].

To further investigate the potential involvement of ZNF671 on HDCA-mediated effects, we designed an adeno-associated virus (AAV) carrying short-hairpin RNA (shRNA) targeting ZNF671 in the Tregs with or without HDCA treatment. Loss of ZNF671 resulted in diminished lipid storage and lesion formation, indicating its crucial role in regulating Treg lipid homeostasis. Notably, HDCA treatment further reduced lipid accumulation and lesion area in both control and ZNF671-deficient Tregs, highlighting a cooperative effect on metabolic regulation (Fig. 6C and D). Functionally, ZNF671 deletion markedly elevated anti-inflammatory cytokines IL-10 and IL-35, potentiating the immunoregulatory profile of Tregs. Consistently, HDCA exerted an additional suppressive effect on cytokine production, especially in the absence of ZNF671 (Fig. 6E and F). Moreover, disruption of ZNF671 promoted Treg migration, which was accompanied by a marked reduction in mitochondrial oxidative phosphorylation (Fig. 6G–I), consistent with the clearly demonstrated role of glycolytic reprogramming in supporting Treg migration, whereby reduced oxidative phosphorylation favors enhanced glycolytic flux and cellular motility [44].

Mechanistically, we further examined the role of ZNF671 in HDCA-mediated STAT5 signaling, given STAT5's well-established function as a central transcription factor in immune regulation and its critical involvement in AS [45]. Immunofluorescence staining revealed that HDCA treatment significantly increased the proportion of pSTAT5-positive cells in the ZNF671 vector group, an elevation that was further pronounced in ZNF671 KO cells, suggesting that the absence of ZNF671 is necessary for optimal STAT5 activation in response to HDCA (Fig. 6J). In alignment with this finding, flow cytometry demonstrated a substantial rise in pSTAT5^+^Foxp3^+^ Tregs following HDCA stimulation and ZNF671 loss, with the inhibitory effects on phosphorylation being most prominent in the absence of ZNF671 (Fig. 6K). These observations collectively implicate ZNF671 as an essential regulator that couples HDCA signaling to STAT5 activation and transcriptional programming of Tregs, thereby linking metabolic cues with immunoregulation. Noted, since our study utilizing pre-existing fully differentiated Tregs, enhanced STAT5 activation likely represents functional modulation of Treg activity, rather than de novo Treg differentiation [46].

## 8 HDCA modulates metabolic pathways and disease-associated signatures in Treg cells

**Fig. 7:**
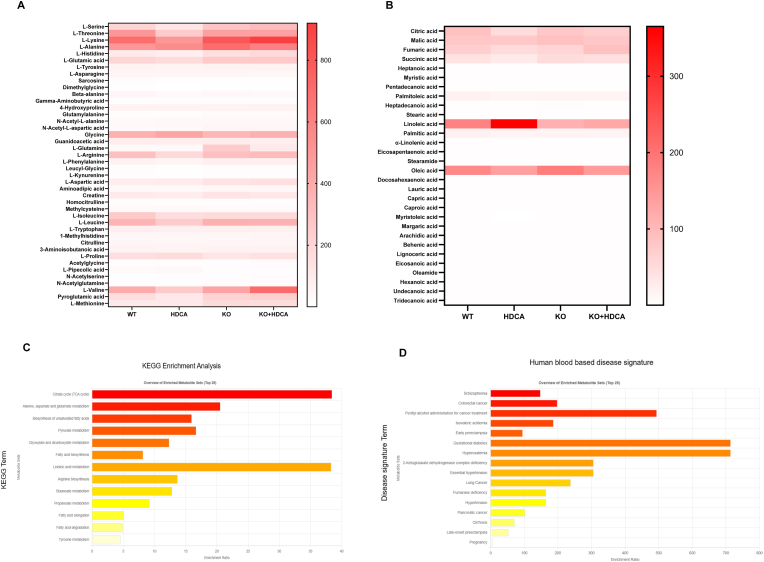

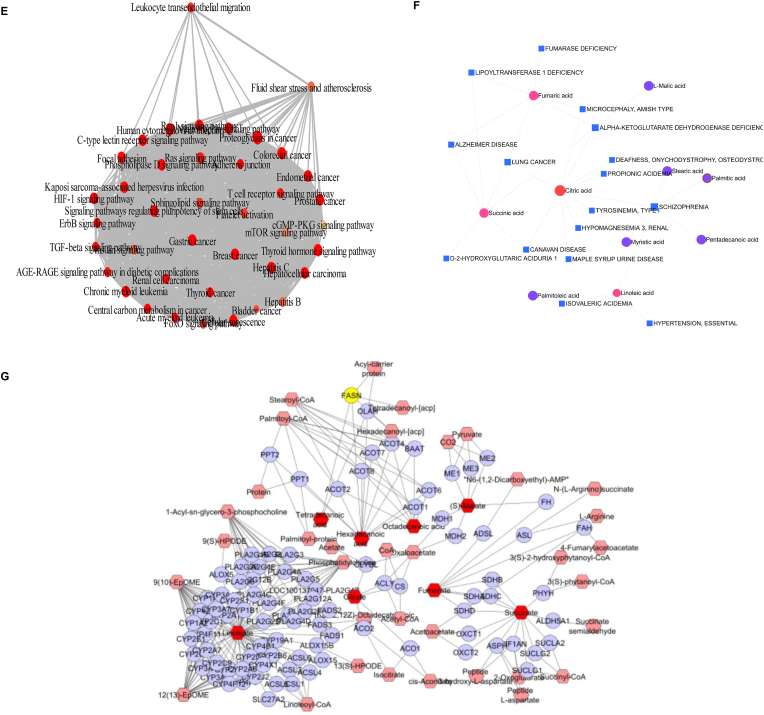
**HDCA regulates amino acid and fatty acid metabolism to improve metabolic balance in atherosclerotic mice. (A**–**B)** Heatmaps showing the relative abundance of (A) key amino acids and (B) fatty acids in Tregs from WT and FXR KO mice ± HDCA. **(C)** KEGG pathway enrichment analysis of altered metabolites, highlighting key pathways such as TCA cycle, unsaturated fatty acid biosynthesis, pyruvate metabolism, and linoleic acid metabolism. **(D)** Disease signature analysis was conducted to assess the enrichment of these key metabolites in human blood. **(E)** Network diagram illustrating significantly enriched KEGG pathways based on differential metabolites after HDCA treatment, highlighting pathways related to leukocyte transendothelial migration, fluid shear stress and AS. (**F)** Metabolite-disease interaction network linking key differential metabolites (red and purple nodes) with corresponding human diseases (blue nodes), illustrating the relationships between altered metabolic profiles and disease signatures. **(G)** Metabolic pathway interaction network showing connections between significantly altered metabolites (red nodes) and related metabolic enzymes (purple nodes), highlighting the functional integration of these metabolites within relevant biochemical pathways.

Expanding upon these findings, we elucidated the impact of HDCA administration and FXR KO on amino acid and fatty acid metabolism ex vivo. The amino acid metabolomic profile revealed significant changes in several amino acids, including serine, lysine, glutamic acid, arginine, valine, leucine, and isoleucine following HDCA treatment. In contrast, FXR KO mice displayed no obvious changes in these amino acids with or without HDCA treatment (Fig. 7A, Fig. S5A). Notely, l-arginine availability influences global metabolic pathways in activated T cells, with decreased levels promoting a shift from oxidative phosphorylation to glycolysis [47]. In addition, the fatty acid profile exhibited a pronounced shift, characterized by a significant elevation in linoleic acid and a pronounced reduction in stearic acid, indicating an improvement in AS (Fig. 7B, Fig. S5B). Pathway enrichment analysis demonstrated that HDCA treatment affected leukocyte transendothelial migration, fluid shear stress and AS, as well as the mTOR signaling pathway, highlighting the close relationship between cell migration and AS (Fig. 7E).

Functional enrichment analyses further highlighted the biological relevance of these metabolic perturbations. KEGG pathway enrichment revealed strong associations with key immune-related pathways, including TCA cycle, biosynthesis of unsaturated fatty acids, and linoleic acid metabolism (Fig. 7C). In parallel, disease signature enrichment analysis based on human blood metabolite signature highlighted essential hypertension, gestational diabetes and early preeclampsia, indicating the clinical implications of the observed metabolic shifts (Fig. 7D). Additionally, integrative network analyses mapped the relationship among specific metabolites, disease phenotypes, and related enzymatic regulators. Visualization of these networks identified that signature metabolites, such as citrate and linoleate, serve as central nodes associated with clinically relevant diseases, while FASN was highlighted as a key regulatory enzyme (Fig. 7F and G). These findings point to the existence of metabolic hubs at the intersection of immune regulation and disease pathogenesis, suggesting that HDCA treatment may profoundly modulate immune homeostasis and attenuate the development of metabolic disorders.

## 9 Discussion

We aimed to investigate the therapeutic effects of FXR pathway regulation by HDCA in AS. Our first observations have shown that HDCA levels are significantly reduced in the blood of AS patients and ApoE−/− mice fed HFD. Consistent with previous reports, patients with coronary artery disease exhibit a significant reduction in the abundance of gut bacterial genes responsible for the biosynthesis of secondary BAs [48]. In parallel, the migration of Treg to arterial lesions in AS patients is positively correlated with the HDCA levels. The treatment of AS mice with HDCA ameliorated the features of arterial plaques, which was accompanied by an increase in Treg migration to the lesion sites without affecting other immune cell subsets. This observation complements and expands on earlier research that underscore the indispensable role of Tregs in constraining AS development, as their depletion significantly accelerates lesion formation [49]. Additionally, HDCA treatment promoted beneficial systemic effects, such as reduction of heart rate, blood pressure and serum cholesterol.

Recent immunological studies have revealed that secondary BA metabolites, including isoDCA and ω-muricholic acid, antagonize FXR transcriptional activity, thereby promoting peripheral Treg (pTreg) differentiation and dampening pro-inflammatory responses [50]. Mechanistically, FOXP3 drives CD4^+^ T cell commitment to pTregs while RORγt alone induces Th17; sequential FOXP3 followed by RORγt yields pTregs with enhanced suppressive capacity [51]. Building on these mechanistic insights, our findings reveal that HDCA administration leads to a significant downregulation of proinflammatory cytokines IL-6 and TNFα and an upregulation of immunosuppressive markers such as IL-10, IL-35, CD73, and CD39 in Tregs. Furthermore, HDCA reduces proportions of FoxP3+IL-17+ and FoxP3+IFN-γ+ cells, reinforcing their suppressive phenotype and limiting plasticity towards proinflammatory states.

Further experiments using Treg FXR KO provided evidence that the immunomodulatory effects of HDCA are strictly FXR-dependent. In vivo analysis confirmed that FXR KO abolished the anti-inflammatory and atheroprotective effects mediated by HDCA, such as reduced lesion size and enhanced IL-10 production. In addition, HDCA treatment promoted glycolysis and reduced both oxidative phosphorylation and FAO in Tregs, which was also abolished in FXR KO Treg. It is well accepted that glycolysis fuels the bioenergetic demands of Treg migration, whereas suppressive activity relies predominantly on oxidative metabolism [52]. The increase on glycolytic pathways is crucial to provide rapid access of energy to the cytoskeleton rearrangement observed during the process of endothelial transmigration [35].

Delving into lipid metabolism, we found that HDCA treatment results in a pronounced remodeling of cellular lipid homeostasis, characterized by decreased acetyl-CoA levels and downregulation of lipogenic gene such as FASN, ACC, SREBP-1c, and SCD1. This lipidomic shift aligns with reports demonstrating that excessive de novo lipogenesis impairs Treg immunosuppressive function and can disrupt their stability during inflammation [53]. Importantly, HDCA increases the proportion of cardioprotective polyunsaturated fatty acids (PUFAs), notably linoleic acid, while reducing pro-atherogenic saturated fatty acids, such as stearic acid. These metabolic changes are associated with enhanced anti-inflammatory Treg function and stabilization of atherosclerotic plaques, likely mediated through improved endothelial barrier integrity and the suppression of lipid-driven inflammation [54]. Moreover, HDCA augments extracellular matrix stability, evidenced by decreased expression of MMP2 and calpain 2, and increased type I collagen, consistent with a more stable plaque phenotype and reduced risk of rupture [55].

We further explored the mechanisms of beneficial effects mediated by HDCA in AS, focusing on FXR and PD-1 pathways. PD-1 is a cell surface receptor expressed on T cells that suppresses their inflammatory activity. Activation of PD-1 enhances glucose metabolism in T cells [56], which suggests a potential link between PD-1 signaling, glucose metabolism and migration in Treg. A potential link between FXR signaling and PD-1 is suggested by the negative correlation in cancer [57], while activation of FXR by GW4064 increases PD-L1 expression in colorectal cells via the MAPK pathway [58]. Our results show that HDCA profoundly downregulates PD-1, and that pharmacological inhibition of PD-1 amplifies HDCA-induced Treg migration and atheroprotection, consistent with reports that PD-1 blockade favors Treg expansion and function in inflammatory contexts [59]. Additionally,PD-1 blockaded enhances glycolytic metabolism and Treg motility, concomitant with activation of ERK, S6K, and RAC signaling pathways. Notably, these effects are critically dependent on mTORC1 signaling, known to be a master regulator of Treg metabolic programming and functional specialization [60].

To elucidate the underlying mechanisms at the transcriptional level, our analysis identified ZNF671 as a key FXR-dependent transcriptional target of HDCA. ZNF671 suppression recapitulates the metabolic and migratory phenotype induced by HDCA, dampening OXPHOS, enhancing glycolysis, and promoting Treg migration to sites of inflammation. ZNF671 deficiency further potentiated HDCA's anti-atherosclerotic effects and increased STAT5 activation, directly linking energetic reprogramming to Treg functional plasticity. This shift in energy metabolism is underpinned by global metabolomic remodeling, whereby HDCA reconfigures amino acid and fatty acid pathways, enriches processes associated with leukocyte transendothelial migration, and fosters a protective lipid signature [61]. Collectively, these data establish ZNF671 as a critical transcriptional effector downstream of FXR, orchestrating a metabolic switch that fuels Treg motility and promotes plaque stability [54].

While this study delineates a novel immunometabolic axis for HDCA in AS, several limitations should be considered when interpreting the findings. First, our clinical cohort, while revealing an associative trend, was of moderate size and lacked stratification by disease severity or data on dietary and gut microbiota factors that influence BA metabolism. Second, the synergistic effect with PD-1 blockade was demonstrated at a single dose combination; future dose-ranging studies are required to define an optimal therapeutic window and safety profile. Third, our mechanistic conclusions are drawn from the ApoE^−/−^ mouse model; validation in complementary models (e.g., LDLr^−/−^ mice) would strengthen their generalizability. Finally, HDCA was administered via intraperitoneal injection to ensure controlled delivery for mechanistic proof-of-concept; translating these findings will necessitate evaluating oral bioavailability and efficacy. Addressing these points in future studies will be crucial for advancing HDCA-based therapeutic strategies toward clinical application.

## 10 Conclusion

In summary, our findings establish a pharmacological role for the endogenous BA HDCA in the context of AS. Mechanistically, HDCA inhibits Treg FXR signaling, activating both glycolytic and migratory pathways. The therapeutic effects of HDCA are possibly mediated though the inhibition of PD-1 and ZN671 pathways, although further studies are necessary to better understand the role of such targets on AS. Our results underscore the therapeutic potential of modulating the FXR pathway and Treg function in the treatment of metabolic diseases, suggesting that the modulation of these pathways could lead to innovative strategies for managing conditions like AS and other cardiovascular diseases.

## 11 Methodology

### Ethical statement

Human blood was obtained from healthy donors according to ethical approval from the clinical research ethnics committee of the First Affiliated hospital, Zhejiang University of Medicine. With informed consent, this study included: 1. Patient group: patients with atherosclerotic cardiovascular disease (ASCVD) confirmed by coronary angiography or CT angiography, including patients with stable angina, non-ST-segment elevation myocardial infarction or peripheral artery disease, and aged ≥40 years; 2. Tissue sample subgroup: atherosclerotic lesion tissue providers who underwent carotid endarterectomy or lower limb artery plaque atherectomy, and normal tissue providers of carotid artery/lower limb artery for non-lesion controls during the same period; 3. Blood sample cohort: consecutively included new ASCVD patients and age/sex matched healthy controls, all of whom were required to complete baseline blood collection and standardized questionnaires; 4. All subjects were required to have traceable clinical follow-up records (≥1 year) and imaging/laboratory review data. Exclusion criteria: 1. Patients with end-stage renal disease, malignant tumors or autoimmune diseases; 2. Patients with acute infection, stroke or myocardial infarction within the past 3 months; 3. Patients with severe cognitive impairment or unable to cooperate with follow-up; 4. The healthy control group should exclude metabolic syndrome or a history of premature cardiovascular disease in first-degree relatives.

### 11.1 Animal experiments

#### 11.1.1 Mouse model

All the animal experiments were reviewed and approved by the ethics committee of Hong Kong Baptist University (REC/20-21/0584) and were conducted in accordance with the WMA Statement on Animal Use in Biomedical Research. To establish the AS model, 8-week-old male ApoE−/− mice on a C57BL/6J background (provided by the Animal Resources Centre, HKBU) with an average initial body weight of 22-25 g were used. Mice were housed under specific pathogen-free conditions with a 12-h light/dark cycle, controlled temperature (22 ± 2 °C) and humidity (50 ± 10%), and provided with standard chow and water ad libitum before dietary intervention. For AS induction, mice were fed a high-fat diet (HFD) for 28 days as previously described [62] (n = 5 per group). After being fed HFD, partial carotid artery ligation was performed to expose the left carotid artery and ligate its three caudal branches, while leaving the superior thyroid artery intac. HDCA treatment (MedChemExpress, NJ, USA) was given at a dose of 10 mg/kg/day for 6 weeks. This dosage was selected based on prior pharmacokinetic studies of conjugated BA [63] and our pilot dose-response experiments, which established its efficacy in elevating serum HDCA to physiological levels sufficient for FXR modulation without observed toxicity. At the end of the treatment period, mice were sacrificed and tissues were collected for quantification of carotid.

### 11.2 Hemodynamic parameter measurement

The blood pressure and heart rate of mice were measured by non-invasive methods. The measurement was performed using a tail artery blood pressure measuring instrument (CODA blood pressure instrument, CODA-HT2 132000). The mice were fixed in a dedicated restraint device to reduce the interference of exercise stress and ensure that the tail was exposed to the sensor of the instrument. The equipment monitors the changes in blood flow in the tail artery through sensors to obtain systolic blood pressure, diastolic blood pressure and mean arterial pressure (MAP). Before the measurement, the mice were given an appropriate adaptation period (30 min) to ensure that they were in a stable state during the measurement. The heart rate was obtained by simultaneously recording the electrocardiogram (ECG) signal. The measurement was performed in a resting state.

### 11.3 Oil Red O staining

We used the Oil Red O staining kit (C0157S, Beyotime). For cells: slowly aspirate the cell culture medium, wash once with PBS; add 4% paraformaldehyde fixative (P0099) or 10% formaldehyde solution for 10 min, rinse twice with PBS. For frozen sections: take out the frozen sections that have been prepared and stored at −20 °C, put them in the section rack to warm up for 5-10 min. According to the number of samples and the volume of staining working solution required for each sample, prepare the Oil Red O staining working solution in a ratio of 3:2 between Oil Red O solution and Oil Red O diluent, mix well, let stand for 10 min, and use within 2 h. For cells: add an appropriate amount of staining solution to cover the cells for 20 s; remove the staining solution, add an appropriate amount of Oil Red O staining solution, and stain for 10-20 min; add an appropriate amount of staining solution, let stand for 30 s, then remove the staining solution and wash with PBS for 20 s; counterstain the cell nucleus with hematoxylin staining solution (C0107); add an appropriate amount of PBS, evenly cover the cells, observe and take pictures under a microscope. For frozen sections: add an appropriate amount of staining solution to cover the sample for 20 s; remove the staining solution, immerse the sections in the staining solution for staining for 10-20 min; remove the Oil Red O staining solution, immerse in distilled water and wash on a shaker for 20 s; counterstain the cell nucleus with hematoxylin staining solution (C0107), observe and take pictures under a microscope.

#### 11.3.1 Isolation and culture of primary CD4^+^CD25^+^ Tregs

Pooled spleen- and lymph node derived CD4^+^CD25^+^ Tregs were obtained from WT mice using an isolation kit purchased from Miltenyi Biotech (Bergisch Gladbach, Germany) and then sorted using a FACSAiraTM Cell Sorter (BD Biosciences, San Jose, CA, USA). Tregs purity was confirmed by FACS analysis and immunostaining with an anti-Foxp3 antibody. Tregs were cultured at 37 °C in an incubator with 5% CO_2_ and 95% humidity in CD3 (1 μg/mL)- and CD28 (5 μg/mL)-coated 24-well plates in the presence of rIL-2 (20 U/mL) for 5 days. For adoptive transfer experiments, Tregs were treated with HDCA (30 μM) or vehicle control for 12 h ex vivo. Following treatment, cell viability was assessed by Annexin V and 7-AAD staining, and Treg stability markers (Helios, Neuropilin-1) and proliferation marker (Ki-67) were analyzed by flow cytometry to ensure that HDCA treatment did not alter Treg survival, identity, or proliferative capacity prior to transfer.

### 11.4 Lentivirus preparation for gene silencing

HEK293T cells were grown in 10 cm cell culture dishes to 70%. For FXR gene knockout, the cells were transfected with lentiviral plasmids encoding Cas9 and FXR-specific single-guide RNA (sgRNA) using the calcium phosphate method. Supernatants were harvested 48 and 72 h after transfection and concentrated 100-fold in an ultracentrifuge. Aliquots were stored at −80 °C. For transduction of Tregs, cells were seeded in six-well plates and cultured in RPMI 1640 to 60%-70% confluence. Lentivirus was added to the cells in the presence of 5 μg/mL Polybrene (107689, Sigma-Aldrich), and the six-well plates were centrifuged at 2300 rpm for 90 min at room temperature, followed by 8 h incubation at 37 °C with 5% CO_2_. Viruses were removed after 24 h; T cells were washed twice with PBS and incubated in complete DMEM (1852730, Life technologies) for 24 h.

### 11.5 Transwell migration assay

Treg migration was assessed using 5.0 μm pore Transwell chambers (3421, Corning). The lower chambers were filled with RPMI 1640 medium containing 100 ng/mL CXCL10 (560-PB, R&D Systems), 100 ng/mL CCL19 (572808, BioLegend), or 100 ng/mL CCL5 (278-TEC-050, R&D Systems). Tregs (1 × 10^5^) in 100 μL serum-free medium were added to the upper chambers. After 3 h of incubation at 37 °C with 5% CO2, the cells that migrated to the lower chamber were collected and counted manually using a hemocytometer. For quantification, cells from four random microscopic fields per well were photographed and counted. Data are presented as the number of migrated cells per field.

### 11.6 Immunofluorescence and immunohistochemistry

Paraffin-embedded tissue sections (4 μm thick) were prepared and subjected to antigen retrieval in 0.01 M citrate buffer (pH 6.0) using a pressure cooker for 3 min. For immunofluorescence, cells were fixed with 4% paraformaldehyde, permeabilized with 0.05% Triton X-100, and blocked with 2% BSA. Samples were incubated with specific primary antibodies: mouse anti-FOXP3 antibody (ab20034, abcam), anti-CD11c antibody (ab254183, abcam), anti-CD45 antibody (ab317446, abcam), anti-CD68 antibody (ab283654, abcam), anti-Gr-1 antibody (ab25377, Abcam), anti-STAT5 (phospho Y694) antibody (ab30645, Abcam), anti-MMP2 antibody (ab86607, Abcam), anti-Collagen I antibody (ab34710, Abcam), anti-PD1 antibody (ab52587, Abcam), anti-FXR antibody (ab129089, Abcam), followed by incubation with Alexa Fluor secondary antibodies for 1 h. Nuclei were counterstained with Hoechst 33342, and fluorescence images were acquired using a confocal microscope (LSM710 or LSM780, Zeiss). For immunohistochemistry, DAB was used for immunohistochemical color development. Tissue sections were incubated with antibodies: rabbit anti–IFN–γ antibody (ab9657, Abcam), mouse anti-FOXP3 antibody (ab20034, Abcam), and rabbit anti-IL-21 antibody (ab5978, Abcam). Staining intensity and the proportion of positive cells were evaluated in 10 random fields at × 40 magnification. The staining index (SI) was calculated as staining intensity multiplied by the proportion of positive cells to assess marker expression.

### 11.7 Immunoblotting analysis

Proteins were detected using the following antibodies: anti-CPT1a antibody (ab234111, abcam), anti-beta actin antibody (ab8226, abcam), anti-PERK antibody (ab229912, abcam), anti-ERK1+ERK2 antibody (ab184699, abcam), anti-S6K1 antibody (ab14708, abcam), anti-S6K1 (phospho T229) antibody (ab5231, abcam), Rac1/2/3 antibody (G-2) (sc-514583, Santa Cruz), anti-Calpain 1 antibody (ab108400, abcam), anti-MMP2 antibody (ab92536, abcam), anti-IL-10 antibody (ab310329, abcam), anti-ZNF671 antibody (HPA046099, Sigma-Aldrich), anti-MAPK6/ERK3 antibody (ab53277, abcam), SIAH1 recombinant rabbit monoclonal antibody (PSH01-80) (MA5-51926, Thermo Fisher), p-Stat1 antibody (pY701.4A) (sc-136229, Santa Cruz), Stat1 antibody (C-136) (sc-464, Santa Cruz), anti-FXR1 antibody (ab155124, abcam), phospho-Raptor (Ser792) polyclonal antibody (PA5-118730, Thermo Fisher), anti-PD1 antibody (ab214421, abcam), SHP-2 antibody (3752S, Cell Signaling Technology), IL-10R antibody (3F9) (sc-53654, Santa Cruz), GAPDH antibody (6C5) (sc-32233, Santa Cruz), rabbit anti-mouse IgG H&L (HRP) (ab6728, abcam), goat anti-rabbit IgG (H + L) highly cross-adsorbed secondary antibody, Alexa Fluor™ Plus 488 (A32731, Thermo Fisher).

### 11.8 Flow cytometry analysis

For surface marker staining, cells were resuspended in 100 μL of staining buffer (PBS with 2%FBS) and incubated with fluorescence-conjugated antibodies at the appropriate dilution as recommended by the manufacturer. Cells were then stained with monoclonal anti-FOXP3-PE antibody (Sigma-Aldrich, SAB4700611); anti-CD4 (mouse) (Sigma-Aldrich, MABF157B); anti-IL-17A (mouse) (BioLegend, 506901); anti–IFN–γ-PE antibody (BioLegend, 502512); anti-STAT5 (phospho Y694)-PE antibody (BioLegend, 432606); anti-CPT1A antibody (Novus Biologicals, NBP1-67375); anti-ZNF671 antibody (Thermo Fisher, PA5-52732) for 30min at 4 °C in the dark. Fluorescence minus one (FMO) control were included for each marker to accurately define the gating boundaries and to control for background fluorescence. Following staining, cells were washed twice with PBS and analyzed by flow cytometry (BD FACSCanto II). Data analysis was performed using FlowJo software (TreeStar).

#### 11.8.1 Allograft rejection and skin graft models in vivo

BALB/c-derived skin was grafted onto vehicle- and antibody-treated C57BL/6 recipients that had received labeled Tregs or no cells 24 h prior. Graft rejection was monitored daily. Some grafts were removed 5 days after transplantation and the presence of labeled Tregs in the indicated tissues (grafts and spleen) was assessed by widefield fluorescence microscopy. The mean number of labeled cells in at least 10 tissue images per animal (n = 12) was examined as described previously [35].

#### 11.8.2 Electron microscopy of resin-embedded cells

For high-pressure freezing suspension, cultured endothelial cells were harvested by filtering and immediately frozen in a high-pressure freezing apparatus (HPF010; Bal-Tec, Balzers, Liechtenstein). For subsequent freeze substitution, the material was kept at −85 °C for 60 h before slowly being warmed to 0 °C for a period of 18 h. Substitution was performed in an AFS freeze substitution unit (Leica, Bensheim, Germany). The sections were poststained with aqueous uranyl acetate/lead citrate, and images were captured with a Hitachi H7650 transmission electron microscope (Hitachi High-Technologies) operating at 80 kV.

#### 11.8.3 In vitro 6-NBDG uptake assay

Freshly isolated Treg cells were washed in PBS and resuspended in glucose-free T cell medium (11879-020, GIBCO) containing various signal antibodies mentioned above and incubated at 37 °C with 5% CO_2_ for 45 min. A final concentration of 400 μM 6-NBDG (N23106, Life Technologies) in glucose-free Treg cell medium was then added to the cells and the cells were further incubated for 10-15 min. Finally, the cells were washed twice with warm PBS and resuspended in flow cytometry buffer and placed on ice. Immediate analysis was performed using flow cytometry to observe fluorescence uptake by Tregs.

#### 11.8.4 Measurement of ECAR and OCR

Real time bioenergetics analysis of the ECAR and OCR of antibody-stimulated Treg cells was performed using an XF analyzer (Seahorse biosciences). Treg cells were cultured in serum-free, unbuffered XF assay medium (102365-100, Seahorse biosciences) for 1 h. Cells were then seeded (6 × 10^5^/well) into seahorse XF24 cell plates for analysis. Perturbation profiling of the use of metabolic pathways by Treg cells was achieved by the addition of oligomycin (1 μM), FCCP (1 μM), Antimycin A (1 μM), rotenone (1 μM), d-glucose (10 mM), 2-Deoxy-d-glucose (2DG, 50 mM; 103020-100 and 103015-100, Seahorse biosciences). Experiments with the Seahorse system was done with the following assay conditions: 2 min mixing, 2 min waiting, and 4-5 min measurement. Metabolic parameters were calculated. Experiments were done in at least triplicate wells.

#### 11.8.5 Liquid chromatography-mass spectrometry (LC-MS) identification

CD4^+^CD25^+^ Treg cells were isolated from mouse arterial tissue by fluorescence-activated cell sorting (BD Biosciences) using anti-CD4-FITC (BioLegend, 100406), anti-CD25-APC (BioLegend, 102012), and anti-Foxp3-PE (BioLegend, 126404) antibodies. Sorted cells (≥1 × 10^6^ per sample) were washed with ice-cold PBS and metabolites were extracted with 200 μL ice-cold methanol (Sigma-Aldrich, 34885) containing 2 μg/mL chlorpropamide as internal standard (Sigma-Aldrich, C4269). Samples were vortexed, incubated at −20 °C, and centrifuged at 14,000×*g* for 15 min at 4 °C. Supernatants were dried under nitrogen and reconstituted in 100 μL 50% methanol. Metabolite profiling was performed using an ACQUITY UPLC system (Waters, USA) with an ACQUITY UPLC HSS T3 column (2.1 × 100 mm, 1.8 μm; Waters, 186003540). The mobile phases were 0.1% formic acid in water (A, Sigma-Aldrich, 53456) and 0.1% formic acid in acetonitrile (B, Sigma-Aldrich, 34851). Samples were analyzed by Q-Exactive Orbitrap mass spectrometer (Thermo Fisher Scientific) in both positive and negative ion modes. Raw MS data were processed using Xcalibur (Thermo Fisher Scientific) and metabolites were annotated by reference to HMDB, KEGG, and Metlin databases. Differential metabolites were determined based on variable importance in projection (VIP) > 1 and p < 0.05 (two-tailed Student's t-test).

#### 11.8.6 Metabolite pathway enrichment analysis

Pathway enrichment analysis was performed on key metabolites—including citric acid, malic acid, fumaric acid, succinic acid, heptanoic acid, myristic acid, pentadecanoic acid, palmitoleic acid, heptadecanoic acid, stearic acid, linoleic acid, and palmitic acid—identified from Treg cells isolated from atherosclerotic plaques of mice treated with or without HDCA. KEGG pathway enrichment and disease signature analyses were conducted using MetaboAnalyst 6.0 (https://www.metaboanalyst.ca/).Additionally, compound–enzyme relationship graph was generated by the MetScape (https://apps.cytoscape.org/apps/metscape) using the software Cytoscape 3.10.3 downloaded from their official website https://cytoscape.org/download.html [64].

## 12 Statistical analysis

GraphPad Prism 10 and SPSS 16.0 statistical software package were used to perform all statistical analysis. P values in most of in vitro and animal experiments were determined by one-way ANOVA and unpaired two-sided Student's *t*-test. Data were presented as mean ± SD (n = 3-5 biological replicates). In all experiments, ∗*p* < 0.05, ∗∗*p < *0.01 and ∗∗∗*p* < 0.001.

## Funding

This work was supported by the National Natural Science Foundation of China (Grant No. 81800248), the General Research Fund (GRF, Grant No. 12101023), and the Research Committee's Startup Grant (Tier 1) for the Academic Year 2020/21 (Grant No. AY2020/21). Additional support was provided by the HKBU Strategic Development Fund (Grant No. SDF 19-1216-P03), the HKBU Start-up Grant for New Academics (Grant No. 163088 RC), and the HKBU Cheung On Tak Endowed Professorship in Chinese Medicine (Cheung On Tak Charity Foundation).

C. Mauro is supported by the British Heart Foundation Senior Basic Science Research Fellowship FS/SBSRF/22/31031 and by UNION-HORIZON-MSCA-DN-2024-111167421.

## CRediT authorship contribution statement

**Feng Yang:** Conceptualization, Data curation, Formal analysis, Methodology, Software, Validation, Writing – original draft, Writing – review & editing. **Wenqiong Huang:** Data curation, Formal analysis, Methodology, Software, Visualization, Writing – original draft, Writing – review & editing. **Zongzhen Meng:** Data curation, Formal analysis, Methodology, Writing – review & editing. **Meijun Liu:** Data curation, Formal analysis, Methodology, Writing – original draft, Writing – review & editing. **Aiping Lyu:** Funding acquisition, Project administration, Resources, Supervision, Writing – review & editing. **Claudio Mauro:** Funding acquisition, Investigation, Project administration, Resources, Validation, Writing – review & editing. **Kenneth C.P. Cheung:** Conceptualization, Formal analysis, Funding acquisition, Investigation, Project administration, Resources, Supervision, Validation, Writing – original draft, Writing – review & editing.

## Declaration of competing interest

The authors declare that they have no known competing financial interests or personal relationships that could have appeared to influence the work reported in this article.

## Data Availability

Data will be made available on request.

## Appendix A Supplementary data

Multimedia component 1 Supplementary Fig. 1. HDCA enhances Treg suppressive marker expression and reduces pro-inflammatory cytokines. (A-B**)** HDCA treatment significantly increases surface expression of CD73 (A) and CD39 (B) on Treg cells compared to UN controls. (**C-D)** Flow cytometric analysis shows that HDCA reduces the proportion of IL-17A+ (C) and IFN-γ+ (D) Treg cells. (**E)** Flow cytometric analysis of CD4^+^Foxp3^+^ Treg cell percentages in the arterial sections with or without CD25 blockade. **(F)** Measurement of serum total cholesterol levels across all groups. Data are presented as mean ± SD (n = 5 biological replicates). Statistical significance was determined by unpaired two-tailed Student's t-test (A–D) or one-way ANOVA with post hoc test (E–F). ∗*P* < 0.05, ∗∗*P* < 0.01, ∗∗∗*P* < 0.001. Supplementary Fig. 2. HDCA inhibits FXR overexpression or GW4064 mediated effects to confer atheroprotective benefits. (A) Representative images of aortic sections stained with Oil Red O in FXR OE mice ± HDCA treatment. **(B)** Representative histological images of aortic sections stained with H&E. **(C)** Immunofluorescence staining for Foxp3 (green) and DAPI (blue) in aortic sections. **(D)** Representative flow cytometry plots and quantification of PKH26-labeled Tregs in aortic plaques and spleen. **(E)** Dose–response analysis of FXR reporter activity upon GW4064 stimulation. **(F)** Dose–response analysis of FXR reporter activity with increasing concentrations of HDCA in the presence of GW4064. **(G)** Quantification of luciferase activity in FXR reporter assays under various treatment conditions. **(H)** Chromatin immunoprecipitation (ChIP)-qPCR analysis shows that GW4064 strongly increases FXR binding to Nr0b2 and CPT1a promoter regions. **(I)** Quantification of F-actin polymerization by phalloidin staining demonstrates that HDCA promotes cytoskeletal remodeling in Tregs. **(J)** Measurement of active RAC1-GTP levels (OD450 nm) in Tregs detected by ELISA. **(K)** Transwell migration assay quantifying migrated Tregs in response to different chemokines under vehicle, HDCA, or HDCA + GW4064 treatments. Data are presented as mean ± SD (n = 3-5 biological replicates). Statistical significance was determined using two-tailed Student's *t*-test or one-way ANOVA. ns, not significant; ∗*P* < 0.05, ∗∗*P* < 0.01, ∗∗∗*P* < 0.001. Supplementary Fig. 3. HDCA enhances plaque stability and facilitates lipid clearance in arterial plaques. (A) Immunofluorescence co-localization of Collagen 1 (red) and MMP2 (green) in arterial tissue sections from Vector, HDCA, FXR KO, and FXR KO + HDCA groups. Nuclei are counterstained with DAPI (blue) in the merged images. (B) Representative transmission electron microscopy images illustrating lysosomes within arterial plaque across the indicated groups. Quantification of lysosomal fluorescence intensity is displayed in the right panel. Data are presented as mean ± SD. ∗*P* < 0.05; ns, not significant. Scale bars: 250 μm (A), 10 μm (B). Supplementary Fig. 4. HDCA modulates Treg migration and metabolic functions via inhibition of PD-1 pathways. (A-B) Linear regression analysis of the association between Sirius red staining area and HDCA-induced inhibition of PD-1 and FXR protein levels in Tregs isolated from plaques. **(C**–**D)** Linear regression analysis was used to evaluate the relationship between fractional shortening and HDCA-induced decrease in FXR and PD-1 protein expression in Tregs isolated from plaques. **(E)** Representative immunofluorescence staining and quantification of DAPI (blue), FXR (green), and PD-1 (red) colocalization in Tregs with or without HDCA treatment (500 cells per group; scale bar, 25 μm). **(F**–**G)** Flow cytometric analysis was used to quantify PD-1 and Raptor expression levels in Tregs following vehicle or HDCA treatment. **(H)** Flow cytometry histograms showing CD4+Foxp3+ Treg percentages under different treatments. **(I**–**J)** Representative bar graphs of extracellular acidification rate (ECAR; mpH/min, left) and oxygen consumption rate (OCR; pMoles/min, right) in Tregs under the indicated treatments. **(K–N)** Flow cytometric assessment of Tregs was conducted to evaluate downstream signaling and cytokine expression under conditions of vehicle control, HDCA treatment, PD-1 blockade, or combined intervention. (K) Median fluorescence intensity (MFI) of phosphorylated ERK (pERK). (L) MFI of phosphorylated S6K (pS6K). (M) MFI of IL-10. (N) MFI of IL-35. Data are presented as mean ± SD (n = 3-5 biological replicates). Statistical analysis was performed using linear regression analysis (A–D), the non-parametric Mann-Whitney *U* test (E–G), and one-way ANOVA followed by post hoc tests (H–N). Statistical significance was defined as ∗*P* < 0.05, ∗∗*P* < 0.01, ∗∗∗*P* < 0.001. Supplementary Fig. 5. HDCA modulates Treg metabolic pathways involving acetyl-CoA, the TCA cycle, amino acid, and fatty acid metabolism. (A) Schematic of glucose metabolism and the TCA cycle, highlighting associated amino acid pathways. Bar graphs present relative levels of branched-chain and other key amino acids across WT, HDCA, FXR KO, and FXR KO + HDCA groups. **(B)** Schematic representation of glycolysis, the TCA cycle, and fatty acid metabolism. Bar graphs depict changes in TCA intermediates and a range of representative fatty acids among groups. The data were compared using Student's t-tests, ∗*P* < 0.05, ∗∗*P* < 0.01, ∗∗∗*P* < 0.001; ns, not significant. Multimedia component 1
