# Supplementary figures and images for: Hyodeoxycholic acid attenuates atherosclerosis by antagonizing FXR and modulating the PD-1/mTORC1 signaling axis
Source: Redox Biol. 2026 Feb 21;92:104096. doi: 10.1016/j.redox.2026.104096 (PMC12968423; doi:10.1016/j.redox.2026.104096)

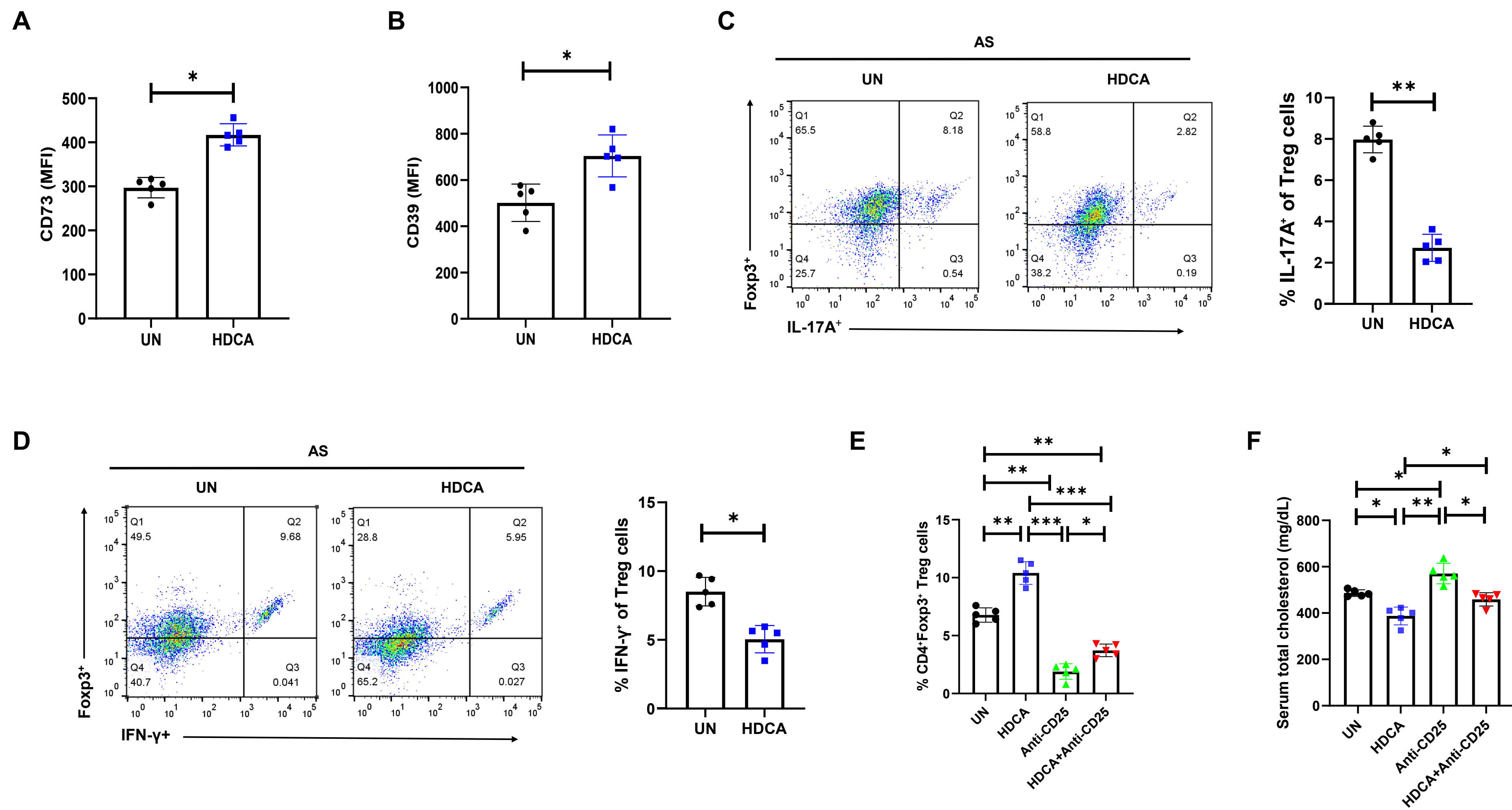

Fig.S1

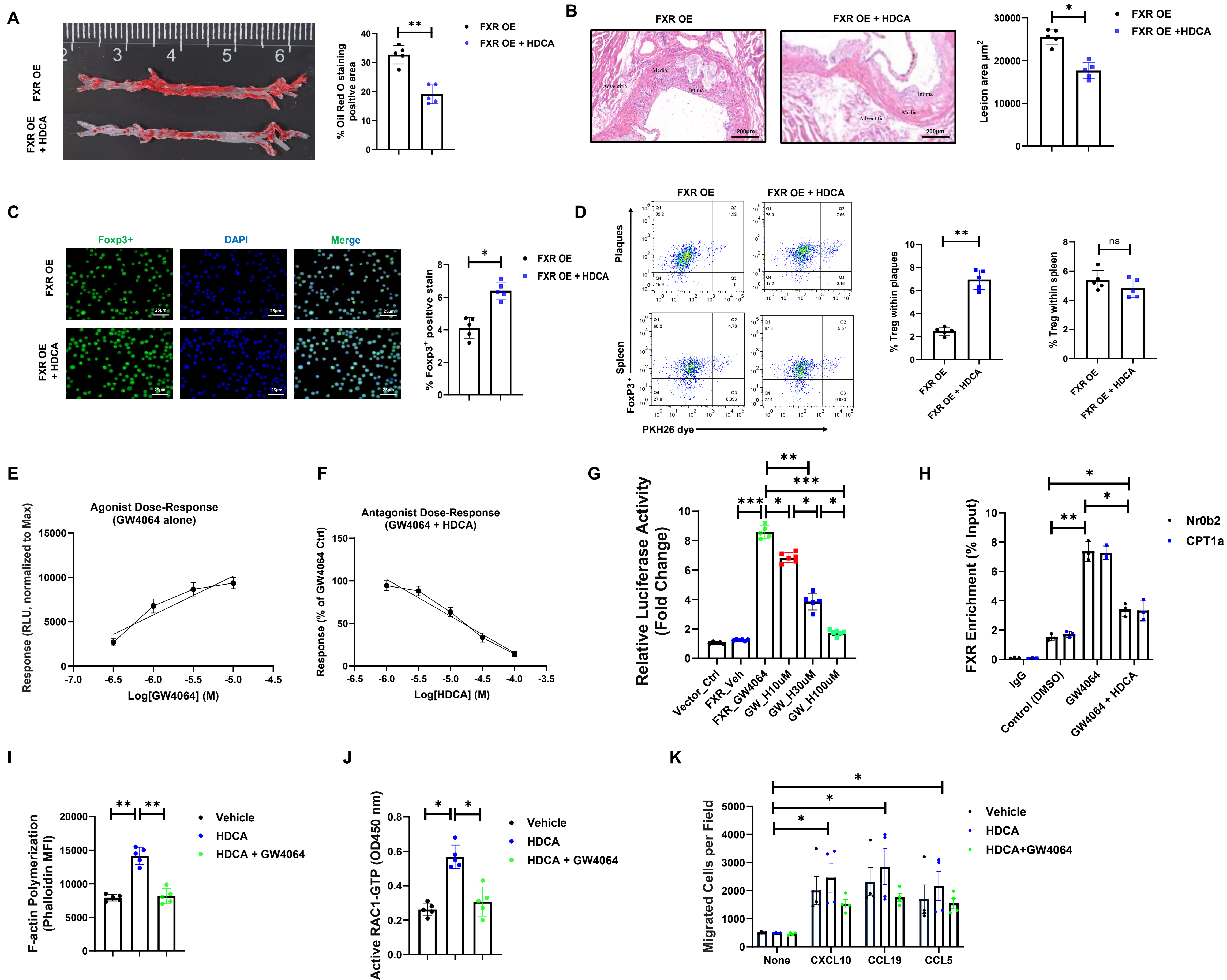

Fig. S2

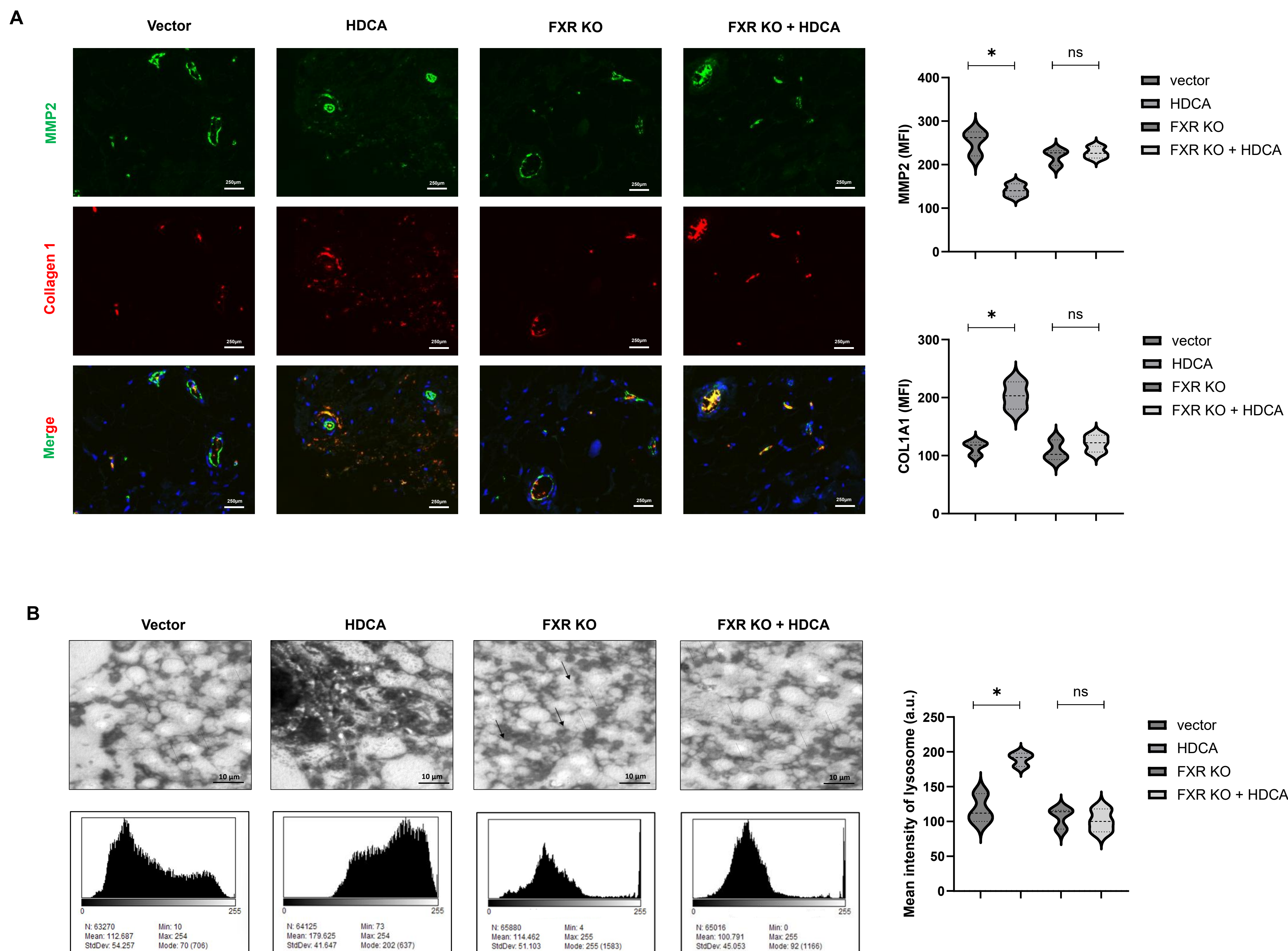

Fig. S3

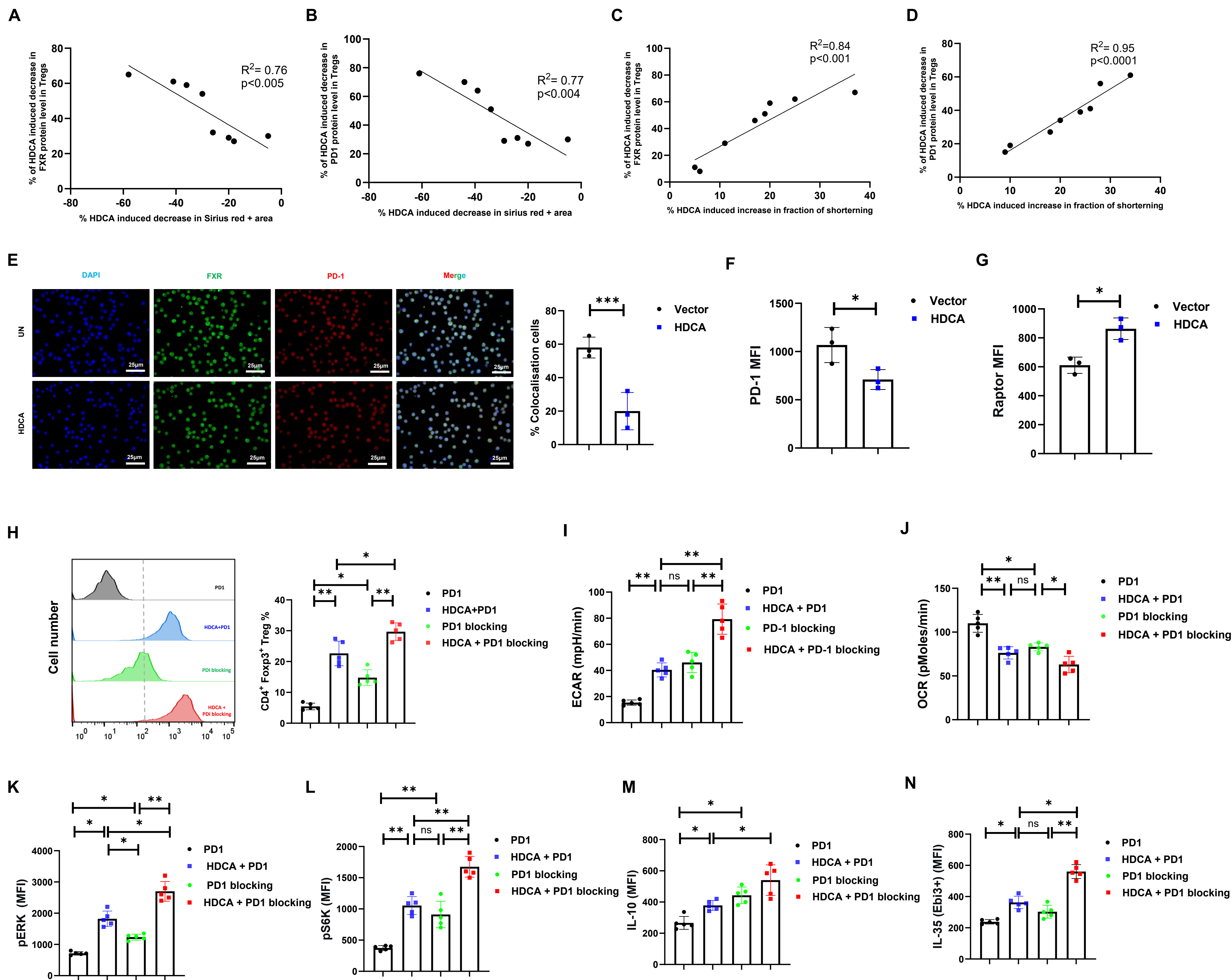

**Fig. S4**

A

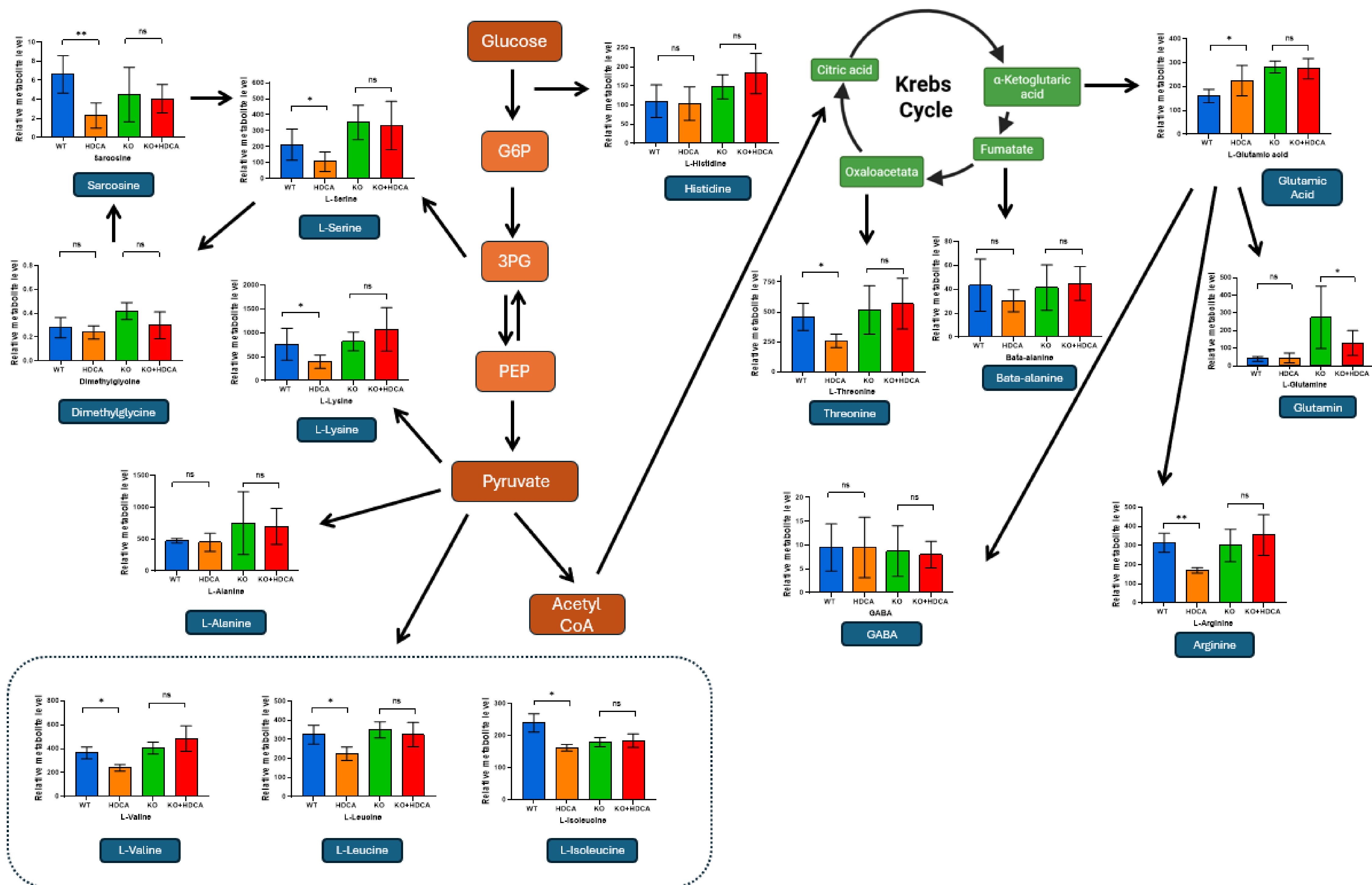

Fig. S5

B

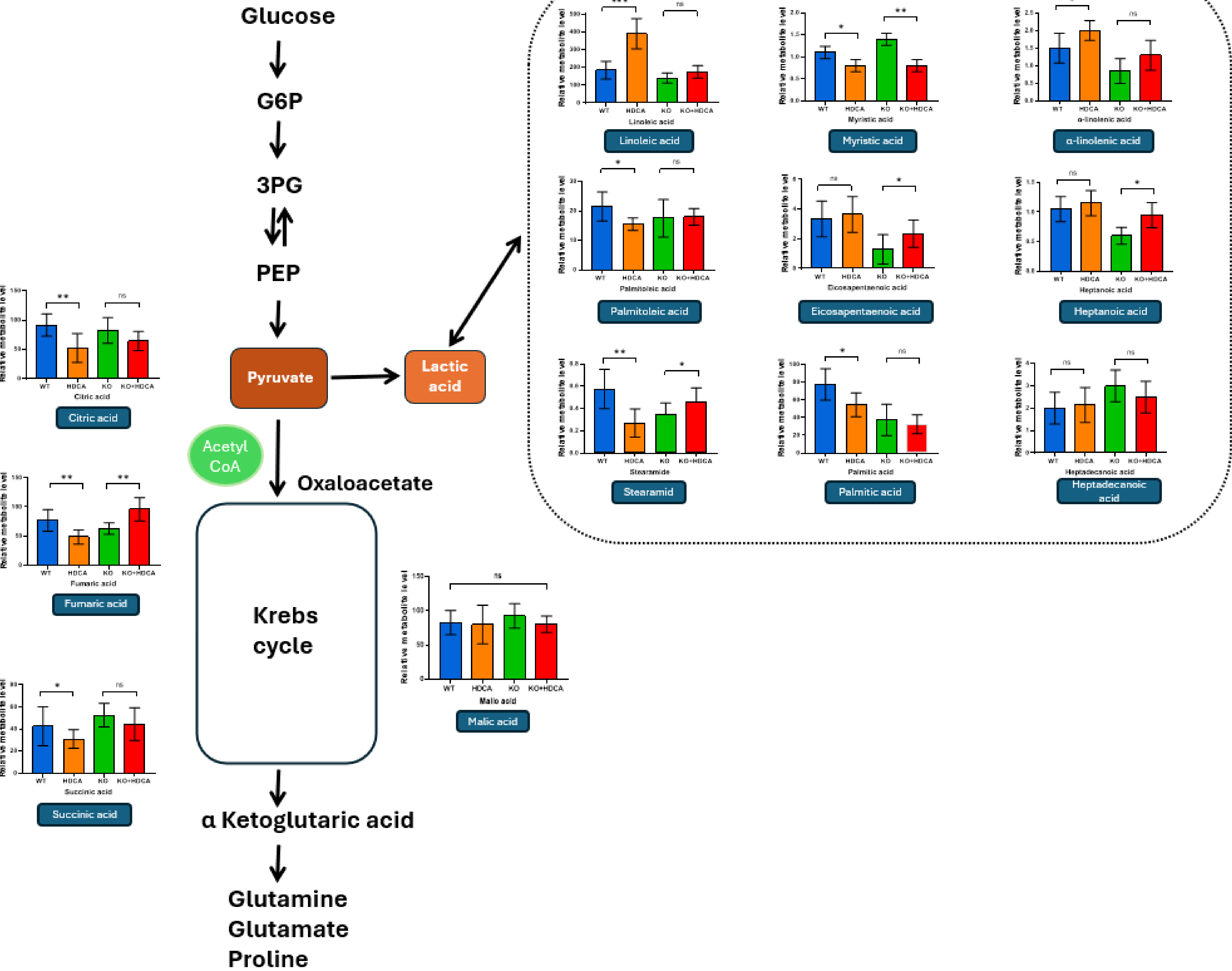

Fig. S5

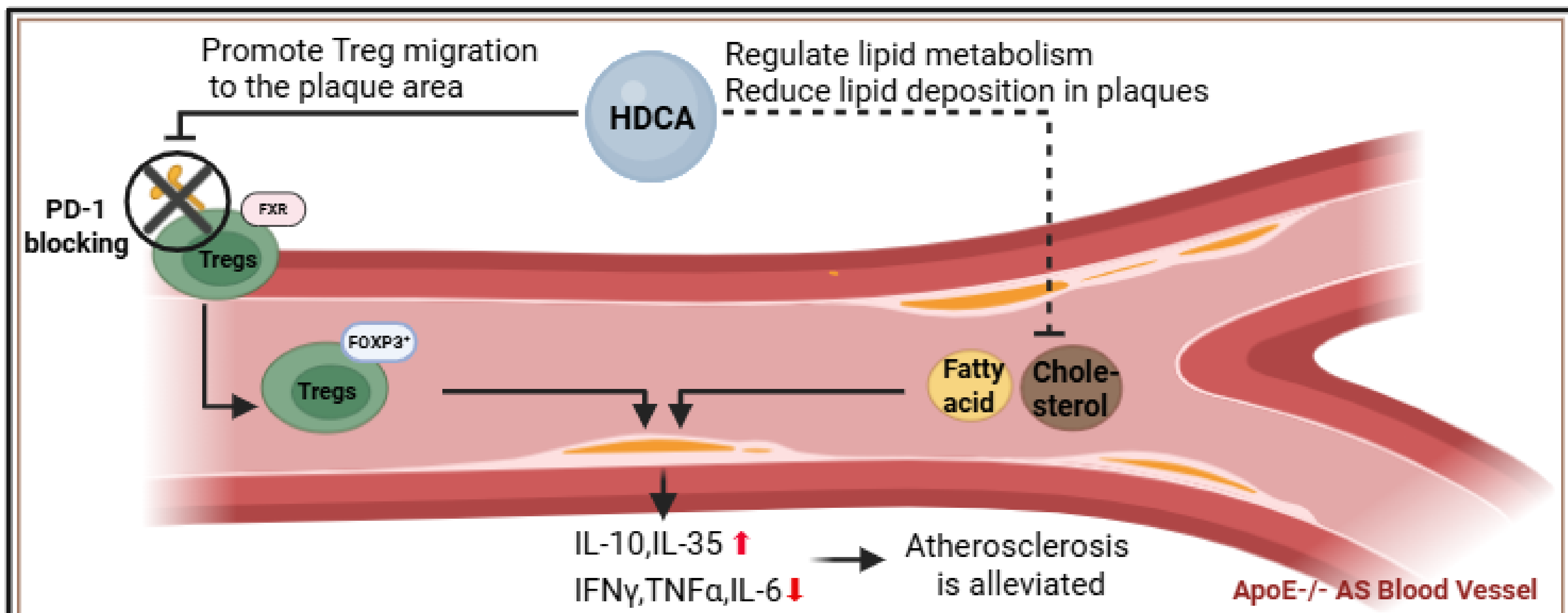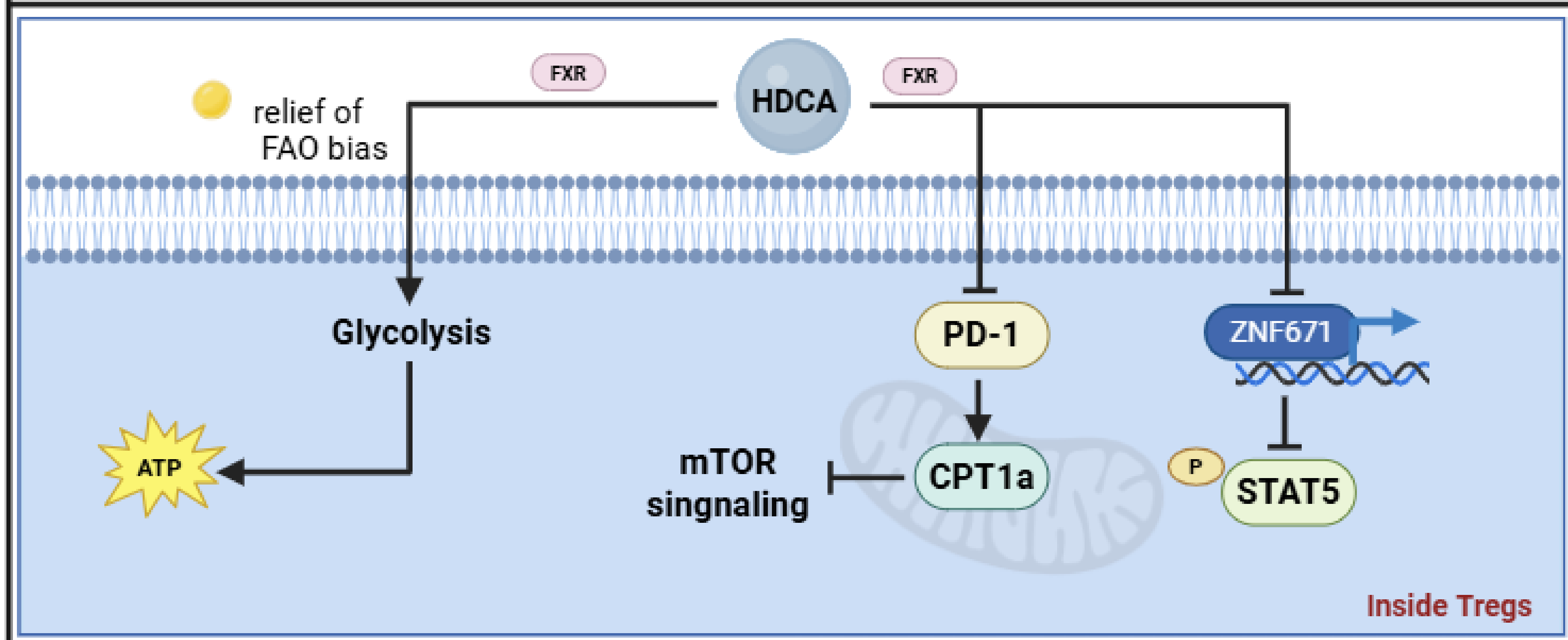

Schematic diagram

Supplement: Multimedia component 1 — Supplementary Fig. 1. HDCA enhances Treg suppressive marker expression and reduces pro-inflammatory cytokines. (A-B) HDCA treatment significantly increases surface expression of CD73 (A) and CD39 (B) on Treg cells compared to UN controls. (C-D) Flow cytometric analysis shows that HDCA reduces the proportion of IL-17A+ (C) and IFN-γ+ (D) Treg cells. (E) Flow cytometric analysis of CD4+Foxp3+ Treg cell percentages in the arterial sections with or without CD25 blockade. (F) Measurement of serum total cholesterol levels across all groups. Data are presented as mean ± SD (n = 5 biological replicates). Statistical significance was determined by unpaired two-tailed Student's t-test (A–D) or one-way ANOVA with post hoc test (E–F). ∗P < 0.05, ∗∗P < 0.01, ∗∗∗P < 0.001. Supplementary Fig. 2. HDCA inhibits FXR overexpression or GW4064 mediated effects to confer atheroprotective benefits. (A) Representative images of aortic sections stained with Oil Red O in FXR OE mice ± HDCA treatment. (B) Representative histological images of aortic sections stained with H&E. (C) Immunofluorescence staining for Foxp3 (green) and DAPI (blue) in aortic sections. (D) Representative flow cytometry plots and quantification of PKH26-labeled Tregs in aortic plaques and spleen. (E) Dose–response analysis of FXR reporter activity upon GW4064 stimulation. (F) Dose–response analysis of FXR reporter activity with increasing concentrations of HDCA in the presence of GW4064. (G) Quantification of luciferase activity in FXR reporter assays under various treatment conditions. (H) Chromatin immunoprecipitation (ChIP)-qPCR analysis shows that GW4064 strongly increases FXR binding to Nr0b2 and CPT1a promoter regions. (I) Quantification of F-actin polymerization by phalloidin staining demonstrates that HDCA promotes cytoskeletal remodeling in Tregs. (J) Measurement of active RAC1-GTP levels (OD450 nm) in Tregs detected by ELISA. (K) Transwell migration assay quantifying migrated Tregs in response to different ch [file mmc1.pdf]

## Slide 1
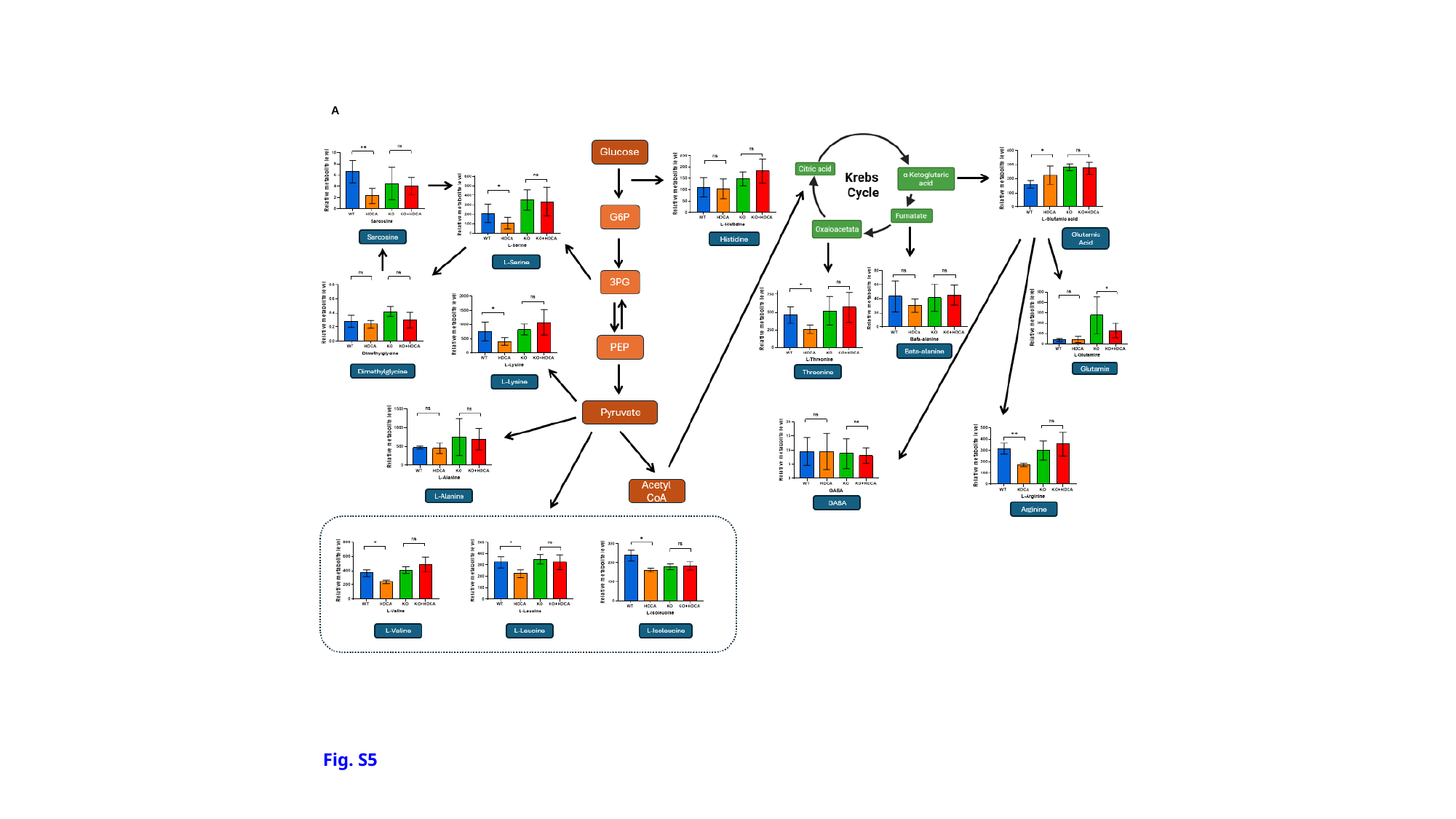

A
Fig. S5

## Slide 2
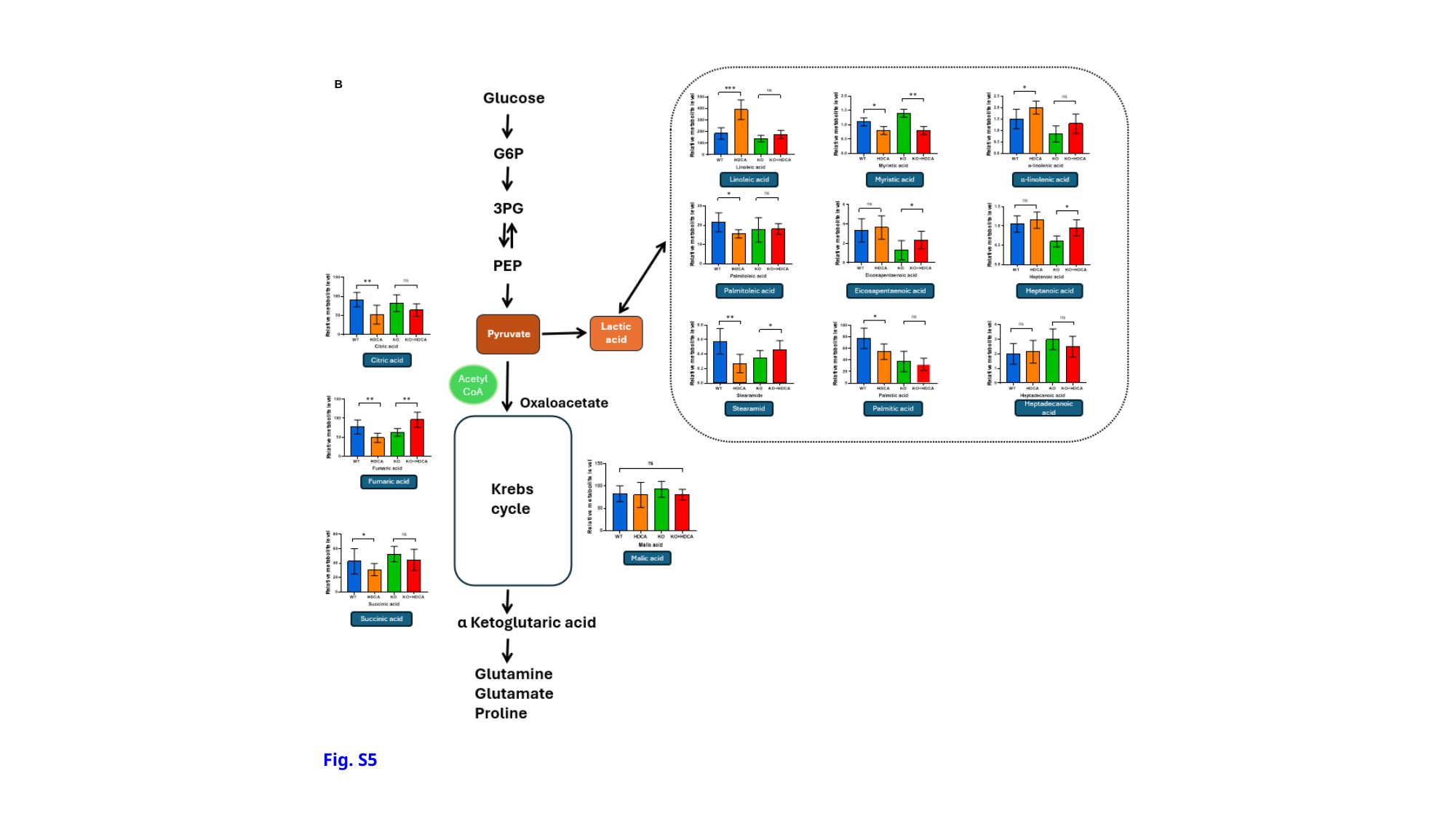

B
Fig. S5

Supplement: Multimedia component 2 [file mmc2.pptx]
